# Secondary Metabolite Profiling, Antiproliferative Evaluation, and Terpenoid Prioritization of Indonesian Cardamom (*Amomum compactum*) Accessions

**DOI:** 10.3390/plants15162538

**Published:** 2026-08-21

**Authors:** Waras Nurcholis, Tamimah Shafwatul Ishlah, Chairunnisa Nur Amanda, Irmanida Batubara, Mohamad Rafi, Rudi Heryanto, Mira Dewi, Heru Cahya Rustamaji, Aryo Tedjo, Bambang Pontjo Priosoeryanto, Wisnu Ananta Kusuma

**Affiliations:** 1 Department of Biochemistry, Faculty of Mathematics and Natural Sciences, IPB University, Bogor 16680, Indonesia; 2 Tropical Biopharmaca Research Center, IPB University, Bogor 16151, Indonesia; tamimah.ishlah@apps.ipb.ac.id (T.S.I.); amandadermawan1@gmail.com (C.N.A.); ime@apps.ipb.ac.id (I.B.); mra@apps.ipb.ac.id (M.R.); rudi_heryanto@apps.ipb.ac.id (R.H.); bpontjo@apps.ipb.ac.id (B.P.P.); 3 Department of Chemistry, Faculty of Mathematics and Natural Sciences, IPB University, Bogor 16680, Indonesia; 4 Faculty of Medicine and Nutrition, IPB University, Bogor 16680, Indonesia; mirade@apps.ipb.ac.id; 5 Department of Informatics, Faculty of Industrial Technology, UPN Veteran Yogyakarta, Yogyakarta 55283, Indonesia; herucr@upnyk.ac.id; 6 Department of Medical Chemistry, Faculty of Medicine, Universitas Indonesia, Jakarta 10430, Indonesia; aryo.tedjo@ui.ac.id; 7 Bioinformatics Study Program, Faculty of Mathematics and Natural Sciences, IPB University, Bogor 16680, Indonesia; 8 School of Data Science, Mathematics, and Informatics, IPB University, Bogor 16680, Indonesia

**Keywords:** *Amomum compactum*, Indonesian cardamom, LC-MS/MS metabolomics, terpenoids, kaur-16-ene, network pharmacology, molecular docking, antiproliferative activity, tumor-derived cell models

## Abstract

This study compared secondary-metabolite profiles, IC_50_-based antiproliferative activity, and relative selectivity of four Indonesian cardamom (*Amomum compactum* Soland. ex Maton) accessions: Bogor white, Bogor red, Sukabumi, and Ciamis. Ethanol and ethyl acetate extracts were evaluated in MCF-7, MCM-B2, and MCA-B1 tumor-derived cells, with Vero cells as a non-tumor-derived reference and profiled by untargeted LC-MS/MS. Putatively annotated metabolites were further assessed using chemometrics, terpenoid-focused network pharmacology, molecular docking, and 50 ns molecular dynamics simulation. Extract IC_50_ values ranged from 0.959 to 2.210 mg mL^−1^ in MCF-7, 0.818 to 2.733 mg mL^−1^ in MCM-B2, and 1.382 to 3.426 mg mL^−1^ in MCA-B1 cells, while Vero values ranged from 2.762 to 4.000 mg mL^−1^. MCF-7 cells generally showed the greatest overall sensitivity, although the lowest individual IC_50_ occurred with Sukabumi ethyl acetate extract in MCM-B2 cells. Ciamis ethyl acetate extract showed higher relative selectivity toward MCF-7 and MCM-B2 cells. LC-MS/MS putatively annotated 36 metabolites, with terpenoid-related compounds predominating. Kaur-16-ene was computationally prioritized based on predicted interactions with *ESR1*, *EGFR*, *SCN5A*, and *CCND1* and short-timescale *ESR1*-complex stability. These findings demonstrate accession- and solvent-associated differences and support kaur-16-ene for further experimental validation.

## 1. Introduction

Breast cancer remains one of the most frequently diagnosed malignancies in women and continues to be a major cause of cancer-related mortality worldwide [1,2]. Although chemotherapy is widely used in breast cancer treatment, its clinical benefits are often accompanied by substantial adverse effects, which may reduce patient tolerance and quality of life [3,4]. These limitations have encouraged the search for plant-derived compounds with potential antiproliferative activity and structurally diverse metabolites for early-stage bioactivity screening. In this context, medicinal plants are increasingly investigated as promising sources of bioactive metabolites with potential value in cancer prevention and therapy [5,6]. In Indonesia, the exploration of local botanical resources continues to yield promising candidates, such as specific extracts demonstrating antiproliferative activity against MCF-7 breast cancer cells [7]. However, early-stage screening using tumor cell models should be interpreted cautiously because extract-level growth inhibition alone does not establish cancer selectivity or safety toward non-tumorigenic cells.

Cardamom (*Amomum compactum* Soland. ex Maton) is a tropical spice crop belonging to the family Zingiberaceae and the genus *Amomum*, with economic importance and an emerging reputation as a source of pharmacologically relevant secondary metabolites [8,9,10]. In Indonesia, *A. compactum* is commonly recognized as Java cardamom and is distinguished from true cardamom (*Elettaria cardamomum*); it has been described as a locally adaptive Indonesian commodity that is widely cultivated and used as an aromatic spice and source of secondary metabolites [11,12,13]. Botanically, Java cardamom is a perennial aromatic herb of the moist tropics, typically cultivated in shaded, humid environments, and valued mainly for its fruit capsules and seeds as spice and herbal materials [14,15]. Morphologically, Java cardamom is a rhizomatous, clump-forming perennial herb with leafy shoots, lateral inflorescences arising from the rhizome, depressed globose fruit capsules, and aromatic polygonal seeds with a white aril [16]. The fruits are typically small capsules of approximately 1–1.5 cm in diameter, and the species grows best under humid tropical conditions with partial shade and well-drained, organic-rich soil [15,16]. Taxonomically, *Amomum compactum* Sol. ex Maton is currently treated as a synonym of *Wurfbainia compacta* (Sol. ex Maton) Škorničk. & A.D.Poulsen; its native range is mainly Sumatra to western Java, while introduced or cultivated occurrences have been reported in several tropical Asian regions [16,17]. Commercially, Java cardamom is valued for its distinctive flavor and aroma, and its dried fruits and seeds are used as culinary spices, medicinal ingredients, traditional preparations, and raw materials for essential oil, flavoring, and aromatic products [15,16,18]. Previous studies have shown that cardamom contains diverse compounds, including terpenoids and phenolic constituents such as phenolic acids and flavonoids, which have been associated with antioxidant, antibacterial, anti-inflammatory, and antiproliferative activities under specific experimental conditions [19,20,21,22,23,24,25]. Several reports have also identified characteristic volatile and semi-volatile constituents of cardamom, such as 1,8-cineole, α-pinene, sabinene, linalool, α-terpineol, nerol, and specific diterpenoids such as ent-kauranes, which have been documented to exert antitumor mechanisms [21,26,27,28,29]. Recent studies on Java cardamom essential oil further support the biological relevance of *A. compactum* fruit-derived volatile fractions, including antibacterial, angiotensin I-converting enzyme inhibitory, antioxidant, and cell-protective activities [12,30]. Most of these reported pharmacological effects are based on in vitro or experimental model systems, whereas in vivo validation and clinical evidence remain limited. In addition to its culinary value, Java cardamom has been used as a spice, herbal material, and essential oil source in Indonesia, and its essential oil has shown experimental antibacterial and angiotensin I-converting enzyme inhibitory activities; however, direct clinical evidence remains limited and its bioactive potential still requires systematic phytochemical and biological validation [30]. These findings indicate that cardamom is a relevant plant source for the identification of metabolites with potential antiproliferative effects.

From a plant science perspective, the chemical composition of cardamom is rarely uniform. Cardamom accessions can differ substantially in essential oil yield and composition, even when grown under comparable conditions, indicating that metabolite accumulation may vary among accessions and is likely influenced by genetic background [27]. In addition, agronomic factors, postharvest handling, processing, and extraction procedures can strongly affect metabolite recovery and chemical profiles in cardamom [21]. These variations are notable because differences in metabolite composition may potentially influence the biological properties of cardamom extracts, including their functional and pharmacological potential [21]. In the Indonesian context, solvent-dependent differences in flavonoid content and antioxidant activity among cardamom accessions have been reported, while extraction conditions have also been shown to significantly affect total terpenoid recovery and cytotoxic activity [23,31]. Such accession-dependent variation is particularly relevant for Java cardamom because secondary-metabolite accumulation in this species has been linked to genetic and agronomic improvement efforts [13]. Nevertheless, despite growing interest in cardamom as a medicinal plant, comprehensive metabolomic characterization of Indonesian cardamom accessions remains limited. In particular, comparative evidence integrating local accession diversity, solvent-dependent metabolite recovery, IC_50_-based antiproliferative assessment, relative selectivity toward tumor-derived cells, and computational compound prioritization is still scarce.

In studies of plant bioactivity, in vitro assays are indispensable for demonstrating measurable biological effects, yet they do not directly reveal which metabolites are most likely to drive the observed responses or which molecular targets may underlie them. Dose–response assays and IC_50_ estimation provide a more quantitative basis for comparing extract-level growth inhibition, while the inclusion of a non-tumor-derived reference cell model allows preliminary assessment of relative selectivity. Metabolomic profiling and in silico analyses, therefore, provide a complementary framework for hypothesis generation and predictive mechanistic inference. Untargeted liquid chromatography–tandem mass spectrometry (LC-MS/MS) allows broad characterization of extract composition and has become a central platform for putative metabolite annotation in plant metabolomics [32,33,34]. Network pharmacology extends this framework by enabling systematic interrogation of disease-associated targets and compound–target–pathway relationships, while molecular docking and molecular dynamics simulation can support the prioritization of candidate metabolites through the evaluation of binding plausibility and complex stability [35,36,37]. Nevertheless, computational approaches such as network pharmacology and molecular docking should be interpreted as hypothesis-generating rather than definitive evidence of target engagement, because their predictive value depends on database completeness, structural assumptions, and subsequent experimental validation [38,39]. Such computational strategies have been successfully applied to screen bioactive compounds from related Zingiberaceae species, demonstrating their usefulness for predictive screening against cancer-associated receptors [40]. The integration of these approaches is therefore well-suited to linking phytochemical variation with predicted breast cancer-related molecular hypotheses, particularly in relation to the human MCF-7 breast cancer model, while extract responses in additional tumor-derived models can provide comparative cellular-background information across distinct cellular backgrounds.

Therefore, this study compared the metabolite profiles of four Indonesian cardamom accessions extracted with ethanol and ethyl acetate, evaluated their IC_50_-based antiproliferative activity and relative selectivity in MCF-7 human breast cancer cells, MCM-B2 canine mammary tumor-derived cells, MCA-B1 canine oral acanthomatous epulis-derived epithelial tumor cells, and Vero non-tumor-derived reference cells, and applied integrated in silico analyses to identify candidate terpenoid compounds and their predicted breast cancer-related molecular targets. The inclusion of MCF-7 is particularly relevant because estrogen receptor-positive breast cancer represents a major clinical subtype, and estrogen signaling is central to breast cancer cell proliferation, survival, and endocrine response [41]. MCM-B2 was included as a canine mammary tumor-derived model established from a benign mixed tumor of the canine mammary gland, providing a comparative mammary tumor-related cellular background [42]. MCA-B1 was included as a canine oral acanthomatous epulis-derived epithelial tumor cell line, characterized by strong keratin immunoreactivity, mild vimentin immunoreactivity, epithelial ultrastructural features, and rapid population doubling, thereby broadening the comparative tumor-derived screening context without classifying it as a breast cancer model [43]. These three tumor-derived cell lines were used as a comparative screening panel across distinct cellular backgrounds rather than as a species-matched or exclusively human breast cancer panel; MCF-7 represented the human breast cancer model, whereas MCM-B2 and MCA-B1 provided comparative canine tumor-derived models for assessing cell-model-dependent extract responses. Vero cells were included as a non-tumor-derived reference model to estimate extract-level relative selectivity, while recognizing that this model does not replace tissue-specific normal breast epithelial controls. Because only one human breast cancer cell line was included, the findings should be interpreted as comparative screening evidence across the tested tumor-derived models, and broader validation will require additional molecularly diverse human breast cancer and tissue-relevant normal cell models. Accordingly, this study provides secondary-metabolite profiling, IC_50_-based bioactivity assessment, and terpenoid-focused computational prioritization framework for future experimental validation of Indonesian cardamom metabolites.

## 2. Results

### 2.1. Antiproliferative Activity, IC_50_ Determination, and Selectivity Index of Ethanol and Ethyl Acetate Extracts

**Figure 1:**
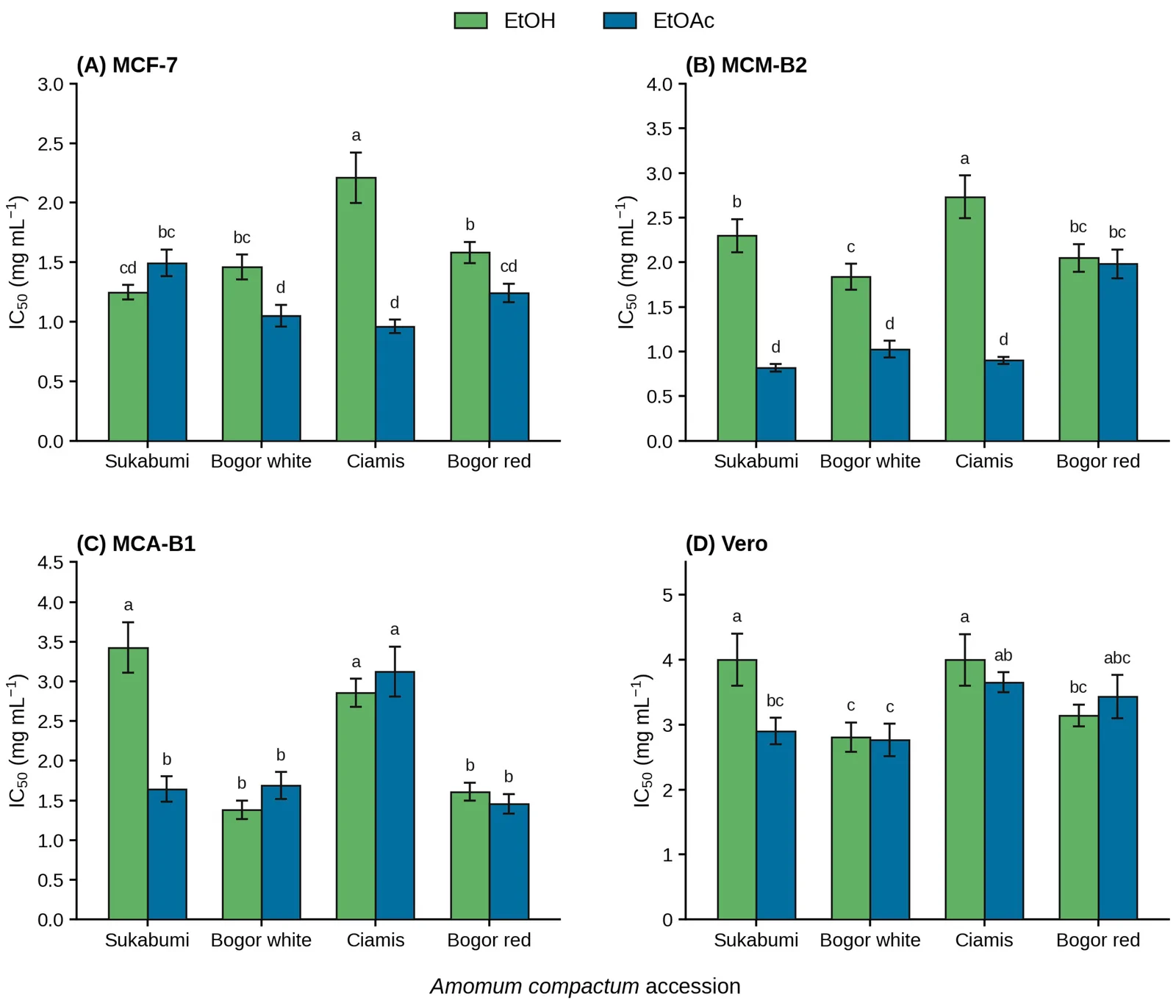
Antiproliferative activity (IC_50_ values in mg mL^−1^) of ethanol (EtOH) and ethyl acetate (EtOAc) extracts from four Indonesian cardamom (*Amomum compactum*) accessions against (**A**) MCF-7, (**B**) MCM-B2, (**C**) MCA-B1, and (**D**) Vero cell lines. Bars represent mean ± SD (n = 3). Different letters (a–d) above the bars within each panel indicate statistically significant differences based on Tukey’s HSD test (*p* < 0.05).

IC_50_ values are presented here as the summarized values across the tested concentrations, rather than the individual inhibition values at each tested concentration. The in vitro antiproliferative activity of four Indonesian cardamom (*A. compactum*) accessions (Sukabumi, Bogor white, Ciamis, and Bogor red) extracted with ethanol (EtOH) and ethyl acetate (EtOAc) was evaluated across three tumor cell models (MCF-7, MCM-B2, and MCA-B1) and non-tumorigenic Vero cells (Figure 1A–D). Overall, antiproliferative responses were significantly driven by extraction solvent, accession type, and their interactions (two-way ANOVA, *p* < 0.05). Medium-polarity EtOAc extracts consistently demonstrated superior growth inhibition compared to EtOH extracts in mammary tumor lines. In MCF-7 human breast cancer cells, IC_50_ values ranged from 0.959 to 2.210 mg mL^−1^, with Ciamis EtOAc displaying the highest potency (0.959 mg mL^−1^; Figure 1A). A more pronounced solvent effect was observed in MCM-B2 canine mammary cells (IC_50_: 0.818–2.733 mg mL^−1^), where Sukabumi EtOAc (0.818 mg mL^−1^) and Ciamis EtOAc (0.900 mg mL^−1^) exhibited the strongest growth inhibition (Figure 1B). Conversely, responsiveness in MCA-B1 canine oral tumor cells was predominantly accession-dependent rather than solvent-driven (IC_50_: 1.382–3.426 mg mL^−1^), with Bogor white EtOH (1.382 mg mL^−1^) and Bogor red EtOAc (1.457 mg mL^−1^) showing the highest activity (Figure 1C).

**Figure 2:**
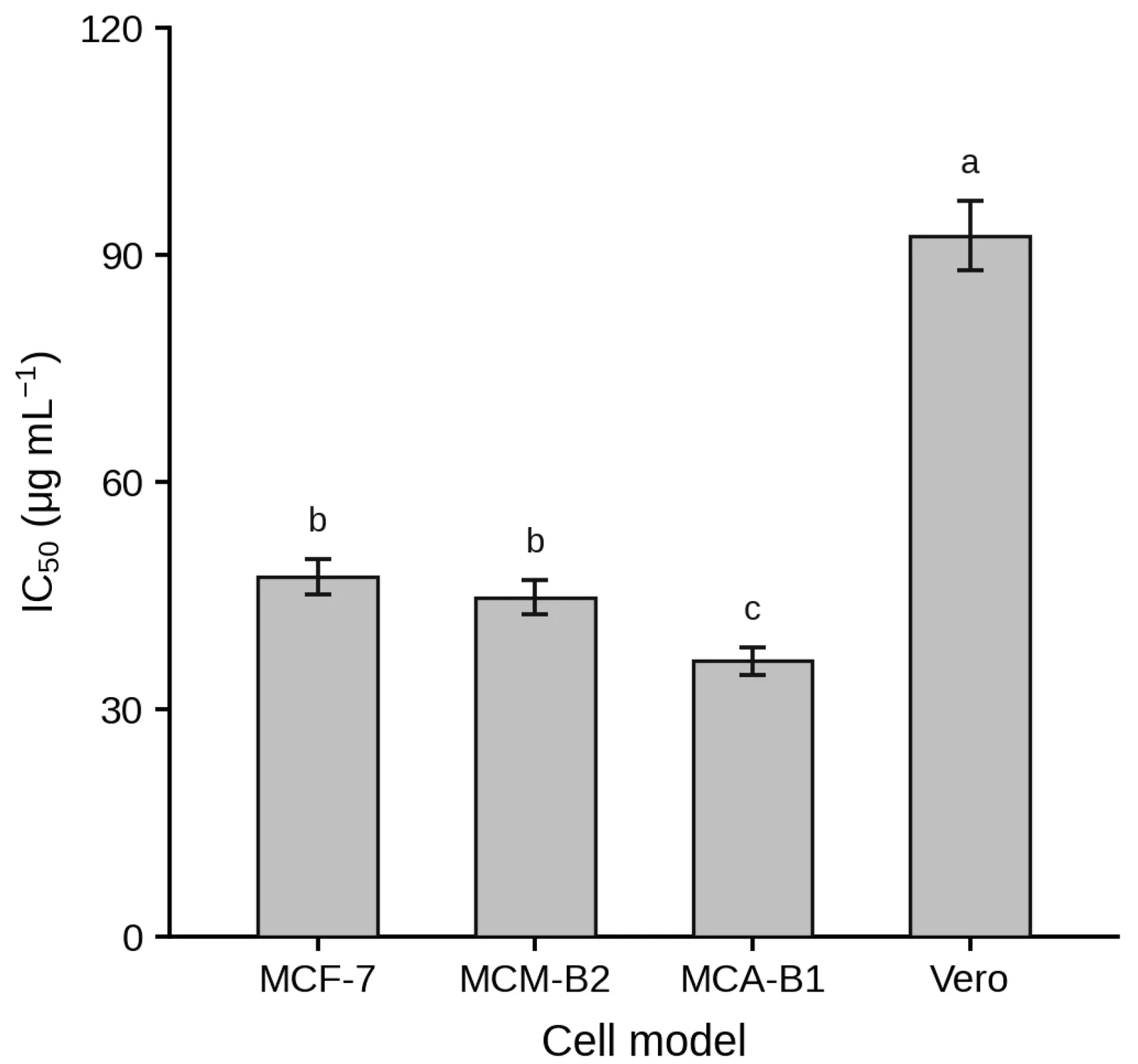
Cytotoxic activity (IC_50_ values in µg mL^−1^) of Doxorubicin as the reference control against MCF-7, MCM-B2, MCA-B1, and Vero cell lines. Bars represent mean ± SD (n = 3). Different letters (a–c) above the bars indicate statistically significant differences based on Tukey’s HSD test (*p* < 0.05).

Basal cytotoxicity in non-tumorigenic Vero cells yielded higher IC_50_ values ranging from 2.762 to 4.000 mg mL^−1^ (Figure 1D), with Sukabumi EtOH and Ciamis EtOH demonstrating the lowest inherent toxicity (both 4.000 mg mL^−1^). Doxorubicin, evaluated as a purified single-compound reference control, exhibited IC_50_ values in the microgram range (36.39–92.50 µg mL^−1^; Figure 2). The milligram-range IC_50_ values of the crude extracts reflect their chemically complex, multi-component nature comprising bulk primary and secondary metabolites, as opposed to a pure commercial chemotherapeutic drug. Cross-referencing tumor and Vero cell IC_50_ values yielded Selectivity Index (SI) values between 1.17 and 4.06 (Table 1). Ciamis EtOAc achieved the highest relative selectivity toward MCF-7 (SI = 3.81) and MCM-B2 cells (SI = 4.06), followed by Sukabumi EtOAc in MCM-B2 cells (SI = 3.55). These findings highlight that semi-polar extraction of specific cardamom accessions (particularly Ciamis and Sukabumi) optimizes both antiproliferative potency and preferential tumor selectivity.

### 2.2. Secondary Metabolite Profiling and Chemometric Analysis by LC-MS/MS

Untargeted LC-MS/MS profiling resulted in the putative annotation of 36 metabolites across the four cardamom accessions (Table 2). These metabolites were assigned to refined chemical classes, including monoterpenoids, monoterpene hydrocarbons, monoterpenoid phenols, sesquiterpene derivatives, diterpene hydrocarbons, fatty acids, phenolic acids, flavonoids, flavonoid glycosides, phenylpropanoids, tannin-related phenolics, coumarins, alkylphenols, phenolic aldehydes, and vitamins/retinoids. Terpenoid-related compounds represented the largest group detected in the extracts. The flavonoid-related group comprised quercetin, quercetin-3β-D-glucoside, quercitrin, hyperoside, and catechin, whereas phenolic-related metabolites were separated into more specific subclasses, including phenolic acids, phenolic aldehydes, alkylphenols, phenylpropanoids, and tannin-related phenolics. Most metabolites were detected at retention times below 10 min, whereas vitamin A, oleic acid, and kaur-16-ene were detected at retention times above 10 min. Quercetin-3β-D-glucoside and hyperoside had the highest molecular weights among the annotated metabolites (464.0945 Da), while tiglic acid had the lowest molecular weight (100.0523 Da). The annotated metabolites were reported together with MSI confidence levels, retention times, precursor ions, product ions, and accession–solvent detection patterns to provide a structured overview of the LC-MS/MS profiling results.

Several metabolites were detected consistently across accessions and extraction solvents, including *p*-cymene, oleic acid, (E,E)-α-farnesene, (2E,6E)-farnesol, and 1,8-cineole. Other metabolites showed more restricted occurrence depending on accession and solvent. These distribution patterns were associated with differences among accession–solvent combinations; however, the present untargeted analysis did not provide absolute metabolite concentrations and therefore does not establish geographic origin as a causal determinant of metabolite abundance. The terpenoid-related compounds were subsequently selected for the bioinformatics analysis described in Section 2.3 because terpenoids represented the predominant chemical group in the LC-MS/MS dataset and are highly relevant to cardamom phytochemistry. This selection was intended as a focused compound-prioritization strategy rather than an exclusion of other bioactive metabolite classes. Non-terpenoid metabolites detected in the extracts, including flavonoids, phenolic acids, phenylpropanoids, tannin-related phenolics, and fatty acids, may also contribute individually or synergistically to the extract-level antiproliferative responses. Therefore, the bioinformatics analysis was designed as a terpenoid-focused hypothesis-generating assessment, while future studies should integrate non-terpenoid metabolites into compound-level target prediction, bioactivity correlation, and experimental validation.

**Figure 3:**
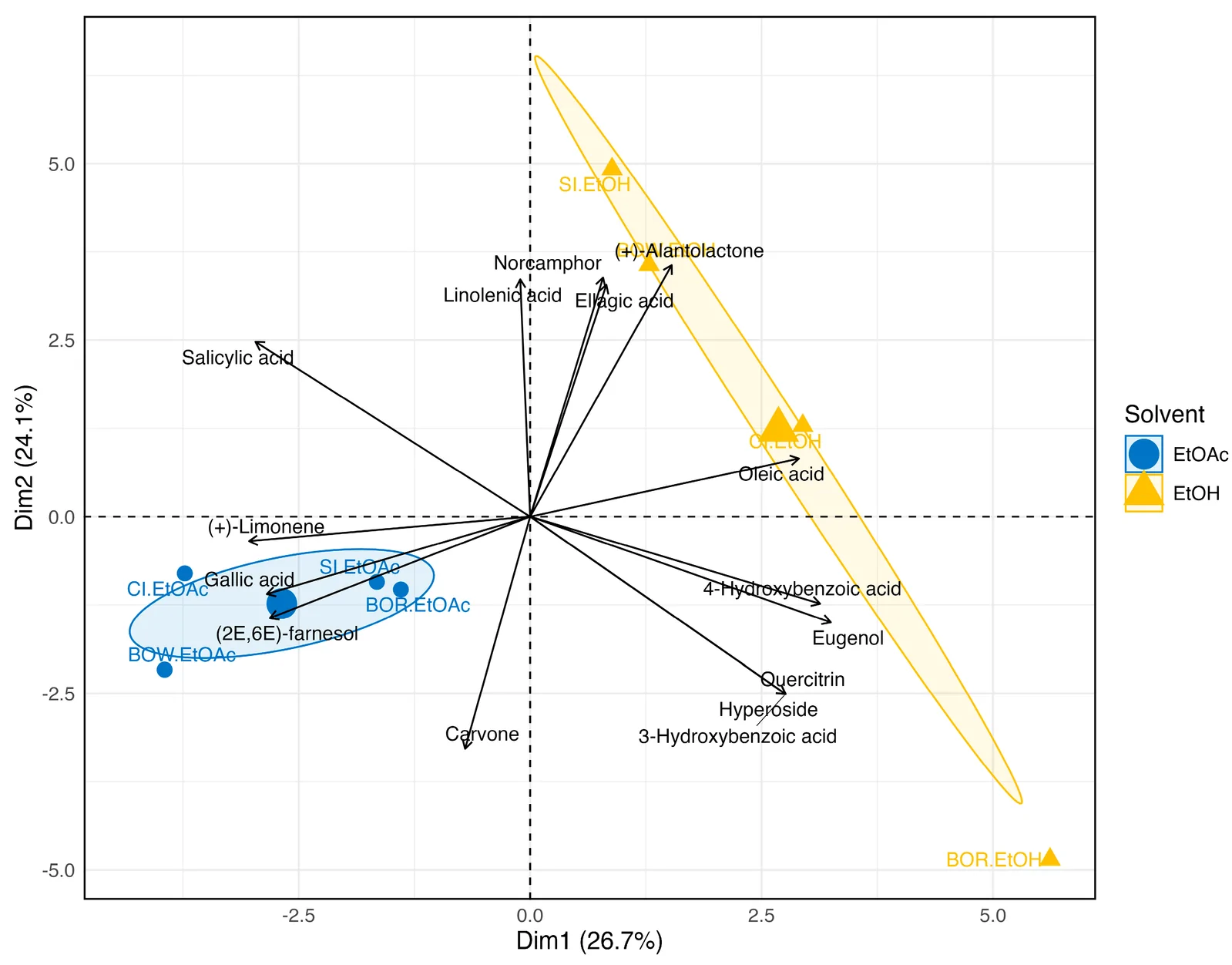
Principal component analysis (PCA) biplot of putatively annotated metabolites from ethanol and ethyl acetate extracts of four Indonesian cardamom (*Amomum compactum*) accessions. Dim1 and Dim2 explained 26.7% and 24.1% of the total variance, respectively. Blue circles represent ethyl acetate extracts (EtOAc), whereas yellow triangles represent ethanol extracts (EtOH). The sample codes indicate accession–solvent combinations: CI, Ciamis accession; BOR, Bogor red accession; BOW, Bogor white accession; SI, Sukabumi accession. Black vectors indicate metabolites contributing to sample discrimination among accession–solvent groups.

Following characterization of the putatively annotated metabolites, untargeted LC-MS/MS data from all eight extracts, consisting of ethanol and ethyl acetate extracts of the four cardamom accessions, were analyzed using principal component analysis (PCA) and hierarchical cluster analysis (HCA) to assess phytochemical variation and accession–solvent-related metabolite patterns among samples. The first two principal components explained 50.8% of the total variance, with Dim1 and Dim2 accounting for 26.7% and 24.1%, respectively. Because the first two dimensions did not capture the full metabolomic variance, the PCA biplot was interpreted as a summary of the dominant two-dimensional separation pattern, while additional variation remained distributed across subsequent components. The PCA biplot showed separation among extracts according to their LC-MS/MS feature profiles, while the vectors identified the metabolites that contributed most to sample discrimination (Figure 3).

**Figure 4:**
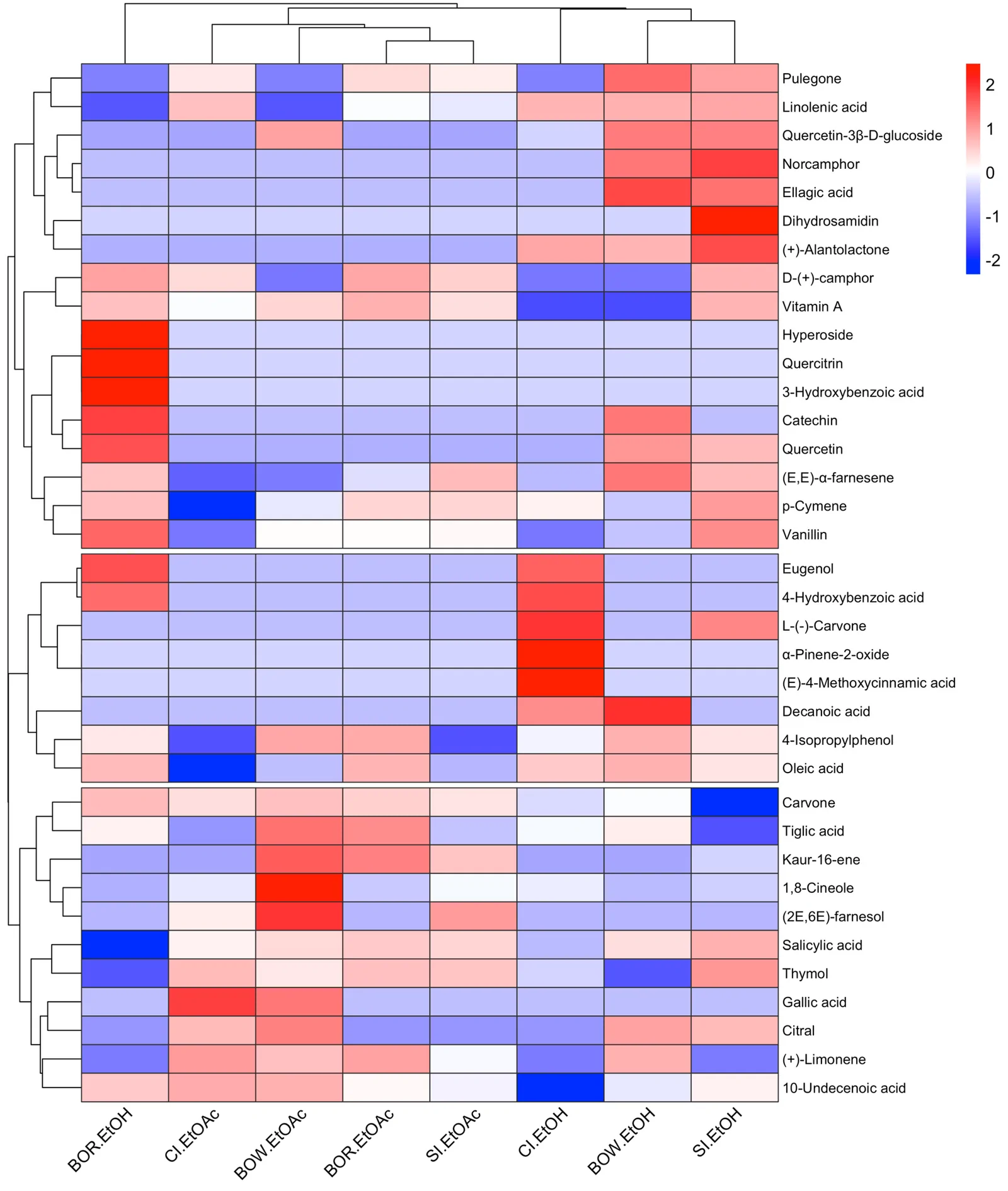
Hierarchical cluster analysis (HCA) heatmap of putatively annotated metabolites from ethanol and ethyl acetate extracts of four Indonesian cardamom (*Amomum compactum*) accessions. Rows represent metabolites and columns represent accession–solvent combinations. Sample codes indicate accession and extraction solvent: CI, Ciamis accession; BOR, Bogor red accession; BOW, Bogor white accession; SI, Sukabumi accession; EtOAc, ethyl acetate extract; EtOH, ethanol extract. Red indicates higher standardized relative abundance, whereas blue indicates lower standardized relative abundance. Dendrograms indicate similarity-based clustering among samples and metabolites, revealing three major metabolite clusters and distinct accession- and solvent-associated phytochemical patterns.

HCA grouped the putatively annotated metabolites into three main clusters (Figure 4). Cluster 1 included compounds such as (+)-alantolactone, L-(−)-carvone, dihydrosamidin, 4-isopropylphenol, *p*-cymene, citral, ellagic acid, norcamphor, (E)-4-methoxycinnamic acid, α-pinene-2-oxide, decanoic acid, 4-hydroxybenzoic acid, and eugenol. Cluster 2 included vanillin, D-(+)-camphor, 10-undecenoic acid, 1,8-cineole, quercetin, catechin, hyperoside, quercitrin, and 3-hydroxybenzoic acid. Cluster 3 included thymol, kaur-16-ene, linolenic acid, salicylic acid, pulegone, vitamin A, (+)-limonene, tiglic acid, gallic acid, (2E,6E)-farnesol, carvone, and (E,E)-α-farnesene. These clustering patterns highlighted distinct differences in metabolite distribution among the ethanol and ethyl acetate extracts of the four accessions and supported the presence of accession- and solvent-associated phytochemical variation.

### 2.3. Terpenoid-Focused Target Prediction, Protein–Protein Interaction, and Functional Enrichment Analysis

**Figure 5:**
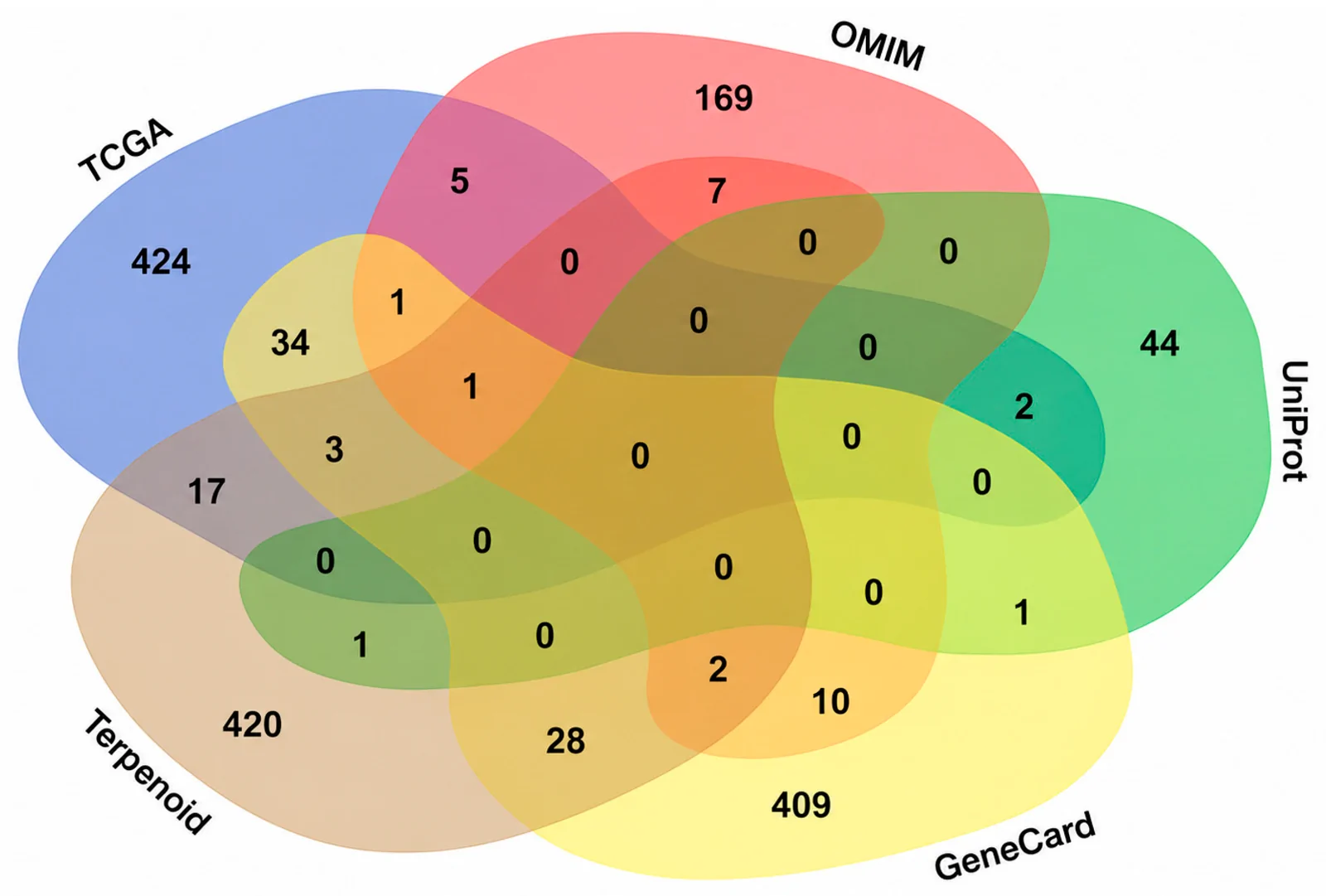
Venn diagram showing the overlap between database-derived predicted target genes of the 15 terpenoid-related compounds and breast cancer-related genes retrieved from OMIM, UniProt, GeneCards, and TCGA. The overlapping genes represent putative candidate targets selected for downstream protein–protein interaction and enrichment analyses, not experimentally confirmed targets.

Based on the LC-MS/MS results, 15 terpenoid-related compounds were selected for a focused bioinformatics analysis. Target prediction of these compounds identified 479 predicted candidate genes. After integration of breast cancer-related genes from OMIM, UniProt, GeneCards, and TCGA and removal of duplicate entries, a total of 1218 breast cancer-associated genes were obtained. Comparison of the predicted terpenoid target set with the breast cancer-related gene set identified 59 overlapping genes as putative breast cancer-associated terpenoid target candidates (Figure 5). These overlapping genes were subsequently used for network construction, hub-gene prioritization, and functional enrichment analysis.

**Figure 6:**
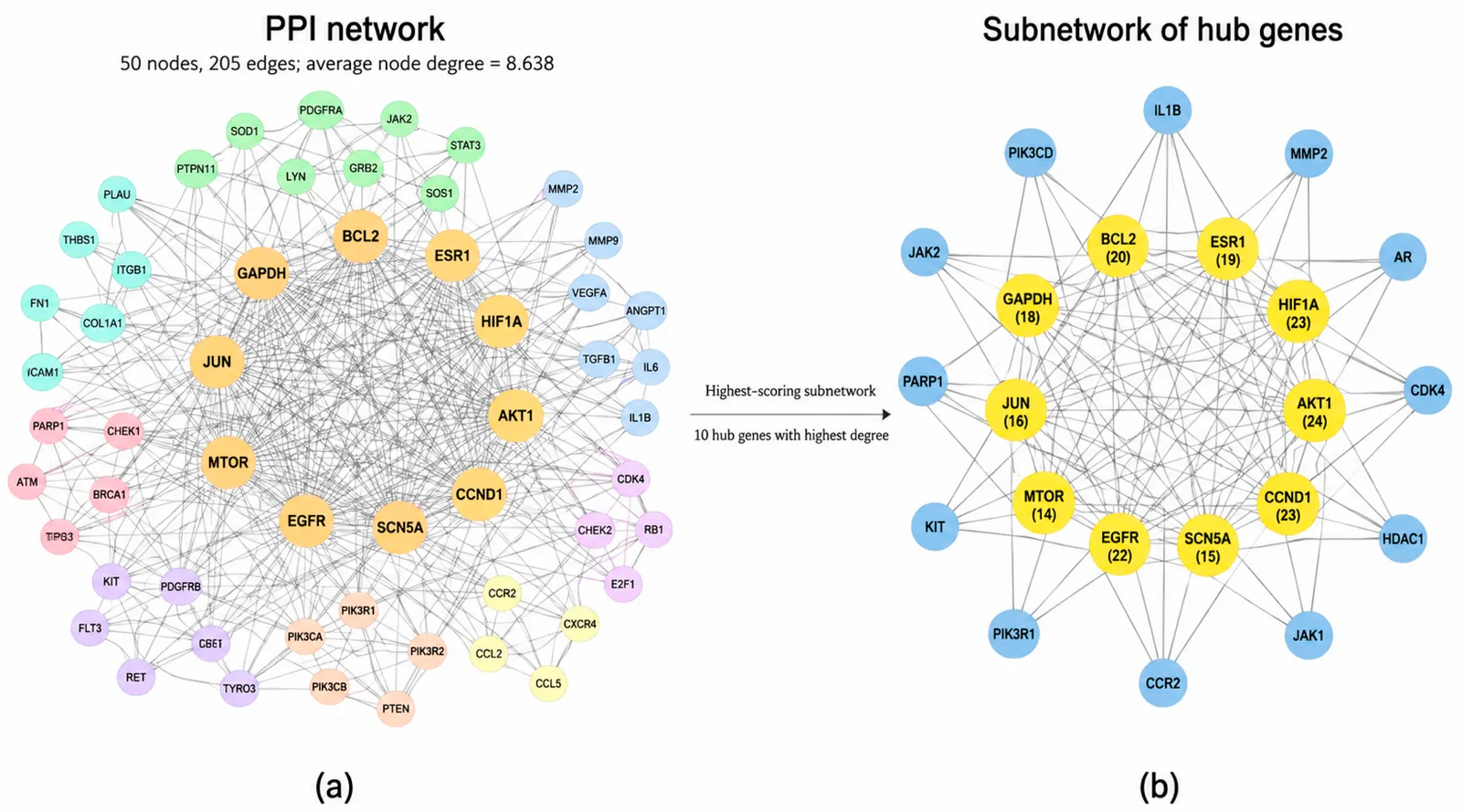
Protein–protein interaction (PPI) network of the overlapping putative target genes. (**a**) Global PPI network generated from STRING. (**b**) Highest-scoring subnetwork extracted from Cytoscape analysis. Yellow nodes indicate the ten predicted hub genes ranked by degree. Node connectivity reflects predicted protein–protein association patterns used for hub-gene prioritization.

The 59 overlapping genes were further analyzed using a protein–protein interaction (PPI) network. STRING analysis generated an interaction network that was subsequently processed in Cytoscape. Network analysis yielded 50 nodes and 205 edges, with an average node degree of 8.638. Cluster analysis identified the highest-scoring subnetwork, from which the ten predicted hub genes with the highest degree values were extracted, namely *AKT1*, *HIF1A*, *CCND1*, *EGFR*, *BCL2*, *ESR1*, *GAPDH*, *JUN*, *SCN5A*, and *MTOR* (Figure 6; Table 3). These hub genes represented highly connected nodes within the predicted interaction network and were therefore prioritized for downstream docking analysis.

**Figure 7:**
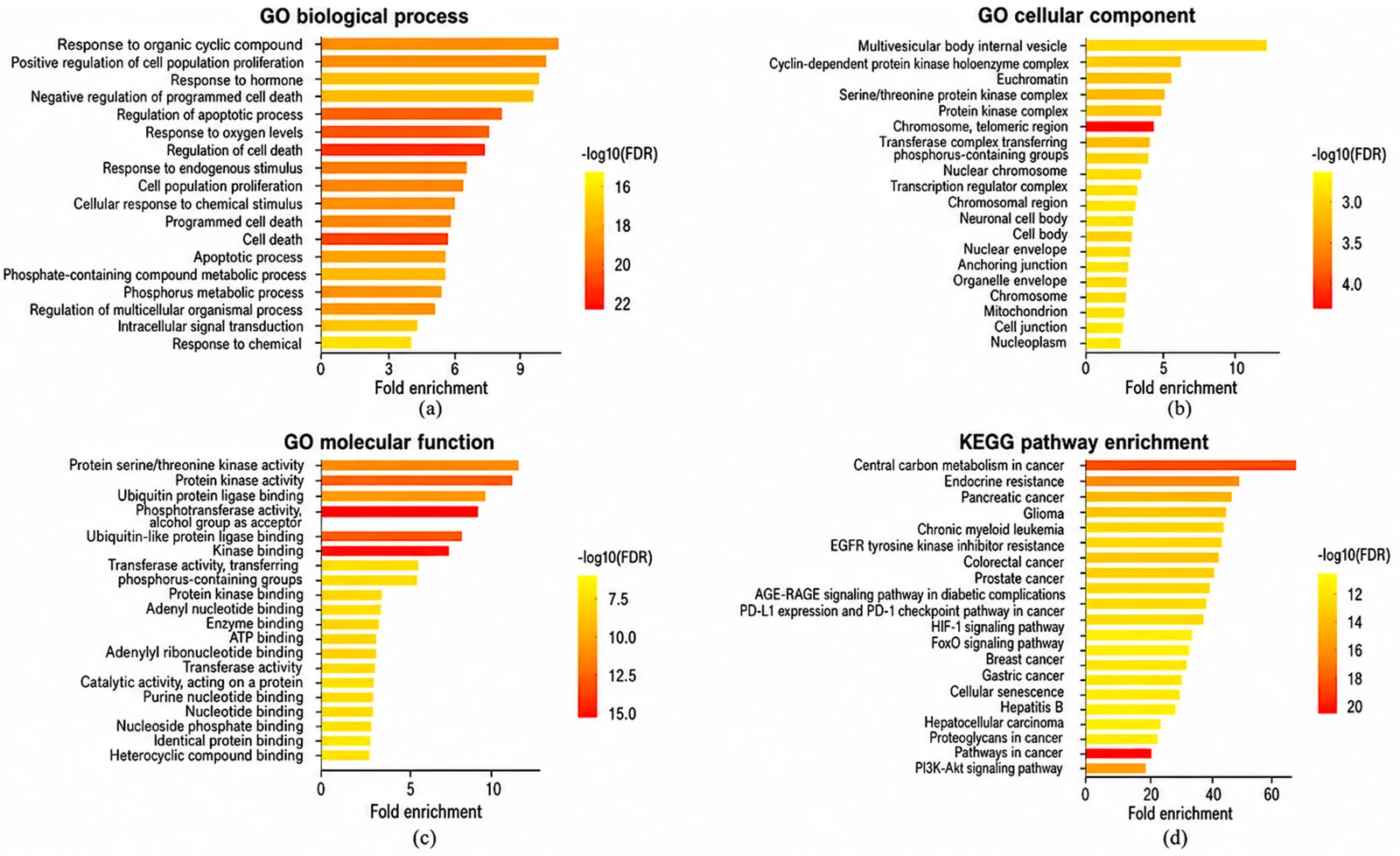
Functional enrichment analysis of the overlapping putative target genes. (**a**) Gene Ontology (GO) biological process; (**b**) GO cellular component; (**c**) GO molecular function; and (**d**) Kyoto Encyclopedia of Genes and Genomes (KEGG) pathway enrichment. Color intensity indicates statistical significance.

Functional enrichment analysis was performed using the hub-associated target genes to examine the enriched Gene Ontology (GO) categories and Kyoto Encyclopedia of Genes and Genomes (KEGG) pathways (Figure 7). In the biological process category, the enriched terms were mainly related to cell proliferation, regulation of cell survival, and cellular response to chemical stimuli. In the cellular component category, the enriched terms were associated with protein complexes involved in transcriptional regulation, phosphorylation, and kinase-related functions. In the molecular function category, the enriched terms were dominated by protein kinase activity, particularly serine/threonine kinase activity.

**Figure 8:**
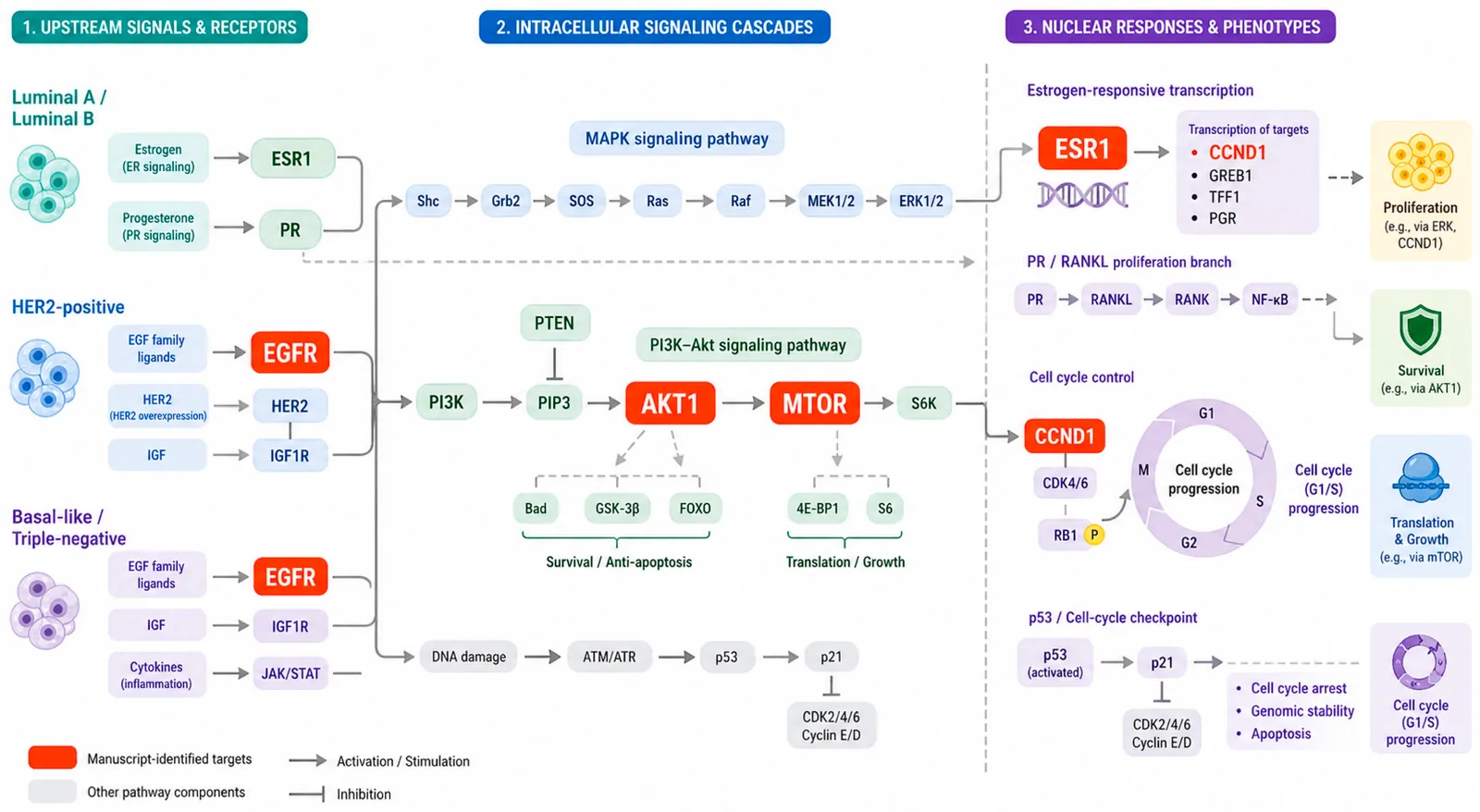
Kyoto Encyclopedia of Genes and Genomes (KEGG) breast cancer pathway showing the predicted terpenoid-related target genes mapped to the pathway. Highlighted nodes represent putative target genes prioritized through the terpenoid-focused target prediction workflow.

KEGG pathway analysis showed enrichment in multiple cancer-related pathways, including breast cancer, pancreatic cancer, colorectal cancer, and gastric cancer. The enriched signaling pathways also included PI3K-Akt signaling, HIF-1 signaling, FoxO signaling, *EGFR* tyrosine kinase inhibitor resistance, PD-L1 expression and PD-1 checkpoint pathway in cancer, and endocrine resistance (Figure 7d). Mapping of the putative target genes onto the KEGG breast cancer pathway showed that *EGFR*, *AKT1*, *MTOR*, *ESR1*, and *CCND1* were directly associated with key components of the pathway (Figure 8). These pathway associations support the prioritization of breast cancer-related signaling nodes for subsequent compound–target docking analysis.

### 2.4. Drug-likeness Screening, Predicted ADMET Profiling, and Molecular Docking of Terpenoid Compounds

To evaluate their drug-likeness and predicted ADMET profiles, 15 terpenoid compounds were selected for drug-likeness screening, absorption, distribution, metabolism, excretion, and toxicity (ADMET) prediction, and molecular docking. Their physicochemical properties and predicted ADMET parameters are summarized in Table 4. Molecular weight ranged from 110.15 to 272.47 g mol^−1^ for the terpenoid compounds, whereas the reference compound doxorubicin had a molecular weight of 543.52 g mol^−1^. Most terpenoids showed LogP values between 1.47 and 4.96, while kaur-16-ene had a LogP value of 5.78. Hydrogen bond donor values ranged from 0 to 1, and hydrogen bond acceptor values ranged from 0 to 2 for all terpenoid compounds. Based on these values, 14 terpenoid compounds complied with Lipinski’s rule-of-five criteria without violation, whereas kaur-16-ene showed one violation because its LogP value exceeded the conventional threshold of 5. In contrast, doxorubicin exceeded the molecular weight, hydrogen bond donor, and hydrogen bond acceptor limits.

The predicted ADMET profiles showed that several low-molecular-weight monoterpenoids, including α-pinene-2-oxide, thymol, pulegone, norcamphor, L-(−)-carvone, D-(+)-camphor, citral, carvone, 1,8-cineole, and (+)-alantolactone, had high predicted gastrointestinal absorption. In contrast, kaur-16-ene, (E,E)-α-farnesene, (2E,6E)-farnesol, (+)-limonene, and doxorubicin showed low predicted gastrointestinal absorption. Kaur-16-ene also showed no predicted blood–brain barrier permeability and was predicted to inhibit CYP1A2, CYP2C19, and CYP2C9, whereas it was not predicted to inhibit CYP2D6 or CYP3A4. Its predicted LD_50_ value was 5000 mg kg^−1^, corresponding to toxicity class 5. Together with the LogP value above 5, these results indicate that kaur-16-ene has a favorable predicted acute oral toxicity class and a highly lipophilic physicochemical profile, suggesting its relevance for subsequent pharmacokinetic and formulation-oriented evaluation.

Docking analysis was performed against ten predicted hub proteins, namely *GAPDH*, *ESR1*, *AKT1*, *EGFR*, *SCN5A*, *BCL2*, *JUN*, *HIF1A*, *MTOR*, and *CCND1* (Table 5). Among the terpenoid compounds, kaur-16-ene exhibited the most favorable predicted binding energies for most targets, including *GAPDH* (−9.1 kcal mol^−1^), *ESR1* (−9.8 kcal mol^−1^), *EGFR* (−7.8 kcal mol^−1^), *SCN5A* (−9.7 kcal mol^−1^), *BCL2* (−7.1 kcal mol^−1^), *HIF1A* (−8.2 kcal mol^−1^), *MTOR* (−10.4 kcal mol^−1^), and *CCND1* (−6.6 kcal mol^−1^). For *AKT1*, kaur-16-ene and vitamin A showed the same binding energy (−8.1 kcal mol^−1^), whereas for *JUN*, kaur-16-ene and (+)-alantolactone showed the same value (−4.5 kcal mol^−1^). Relative to the doxorubicin reference compound under the applied docking protocol, kaur-16-ene showed more favorable predicted docking scores for *ESR1*, *EGFR*, *SCN5A*, and *CCND1*. Doxorubicin was included as a reference compound because it was used as the in vitro positive control; however, it is not a target-specific ligand for *ESR1*, *EGFR*, *SCN5A*, or *CCND1*. Doxorubicin showed lower binding energies for *AKT1*, *BCL2*, *JUN*, *HIF1A*, and *MTOR*, while the same binding energy was observed for *GAPDH* (−9.1 kcal mol^−1^). Overall, kaur-16-ene demonstrated the strongest predicted docking profile among the terpenoid compounds across the selected protein targets and was therefore selected for detailed protein–ligand interaction analysis.

### 2.5. Protein–Ligand Interaction Analysis

**Figure 9:**
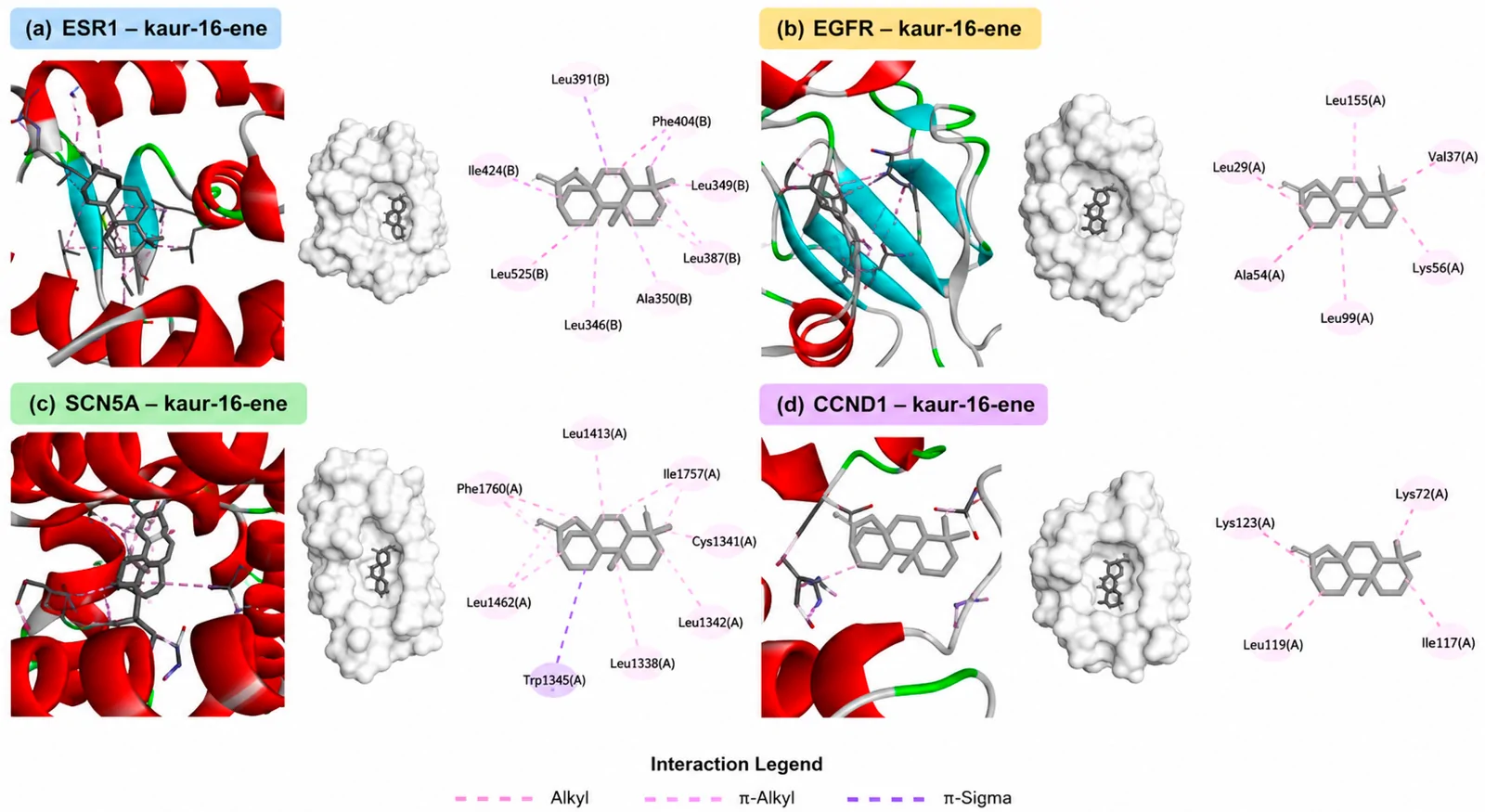
Predicted protein–ligand interaction profiles of kaur-16-ene with selected breast cancer-related hub proteins obtained from molecular docking analysis: (**a**) *ESR1*, (**b**) *EGFR*, (**c**) *SCN5A*, and (**d**) *CCND1*. Each panel shows the predicted three-dimensional binding pose, surface representation of the ligand-binding pocket, and two-dimensional interaction map. The interaction profiles were dominated by hydrophobic contacts, including alkyl and π–alkyl interactions, with an additional π–sigma interaction observed in the *SCN5A*–kaur-16-ene complex. These interaction maps summarize the predicted binding poses and dominant contact types observed in the docking analysis.

Representative predicted protein–ligand interaction profiles of kaur-16-ene with *ESR1*, *EGFR*, *SCN5A*, and *CCND1* are shown in Figure 9, and their full interaction profiles are provided in Table S1. These four targets were selected based on the docking results in Table 5, where kaur-16-ene showed more favorable predicted docking scores than the doxorubicin reference compound for *ESR1*, *EGFR*, *SCN5A*, and *CCND1*. An interaction analysis was performed to characterize the predicted binding poses and dominant interaction types of kaur-16-ene within the selected protein pockets.

In the *ESR1*–kaur-16-ene complex, the interaction was dominated by alkyl and π-alkyl contacts involving Leu391(B), Phe404(B), Leu349(B), Leu387(B), Ala350(B), Leu346(B), Leu525(B), and Ile424(B) (Figure 9a). No hydrogen bond was observed in this complex, indicating that the predicted *ESR1*–kaur-16-ene binding pose was mainly stabilized by hydrophobic contacts.

In the *EGFR*–kaur-16-ene complex, the interaction was dominated by alkyl and π-alkyl contacts involving Ala54(A), Leu29(A), Leu155(A), Val37(A), Lys56(A), and Leu99(A) (Figure 9b). Consistent with the hydrophobic character of kaur-16-ene, no conventional hydrogen bond was observed in the predicted *EGFR* binding pose.

In the *SCN5A*–kaur-16-ene complex, a π-sigma interaction was observed with Trp1345(A), together with alkyl and π-alkyl contacts involving residues such as Phe1760(A), Leu1462(A), Leu1413(A), Ile1757(A), Cys1341(A), Leu1342(A), and Leu1338(A) (Figure 9c). These interactions suggest that the predicted *SCN5A* binding pose was primarily governed by hydrophobic and π-associated contacts.

In the *CCND1*–kaur-16-ene complex, the interaction was dominated by alkyl and π-alkyl contacts, particularly with Lys72(A), Ile117(A), Leu119(A), and Lys123(A) (Figure 9d). No conventional hydrogen bond was detected in this interaction profile, which is consistent with the nonpolar diterpene hydrocarbon structure of kaur-16-ene. Overall, the interaction maps indicated that kaur-16-ene binding to the four selected proteins was mainly associated with hydrophobic contacts rather than polar interactions.

### 2.6. Molecular Dynamics Simulation of ESR1 Complexes

**Figure 10:**
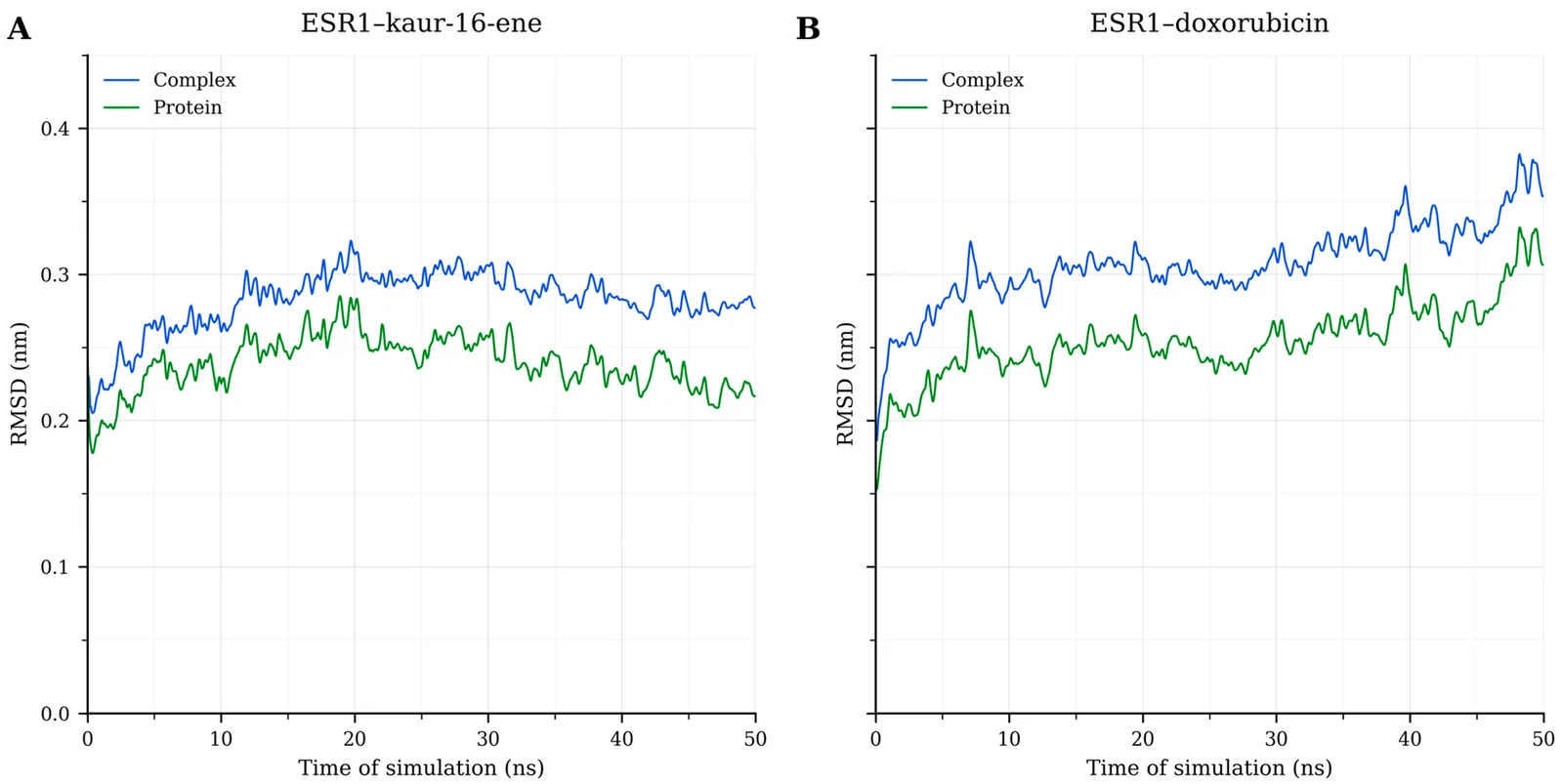
Root mean square deviation (RMSD) profiles of *ESR1* complexes during 50 ns molecular dynamics simulation. (**A**) *ESR1*–kaur-16-ene complex. (**B**) *ESR1*–doxorubicin reference complex. Blue lines represent the protein–ligand complex, whereas green lines represent the protein backbone. RMSD profiles describe time-dependent conformational deviation over the simulated trajectory.

Molecular dynamics simulations were performed for the *ESR1*–kaur-16-ene and *ESR1*–doxorubicin complexes over a 50 ns simulation period to computationally assess their short-timescale conformational behavior. The root mean square deviation (RMSD) profiles are shown in Figure 10. In the *ESR1*–doxorubicin reference system, the complex RMSD increased from approximately 0.18 nm to around 0.28 nm during the initial phase and then fluctuated up to 0.35–0.40 nm toward the end of the simulation. The corresponding protein RMSD ranged from approximately 0.20 to 0.33 nm. In the *ESR1*–kaur-16-ene system, the complex RMSD ranged from approximately 0.25 to 0.31 nm after equilibration, whereas the protein RMSD ranged from approximately 0.18 to 0.26 nm. These RMSD profiles indicate that the *ESR1*–kaur-16-ene complex maintained a comparatively narrower deviation range during the simulated period, suggesting stable short-timescale conformational behavior within the applied trajectory conditions.

**Figure 11:**
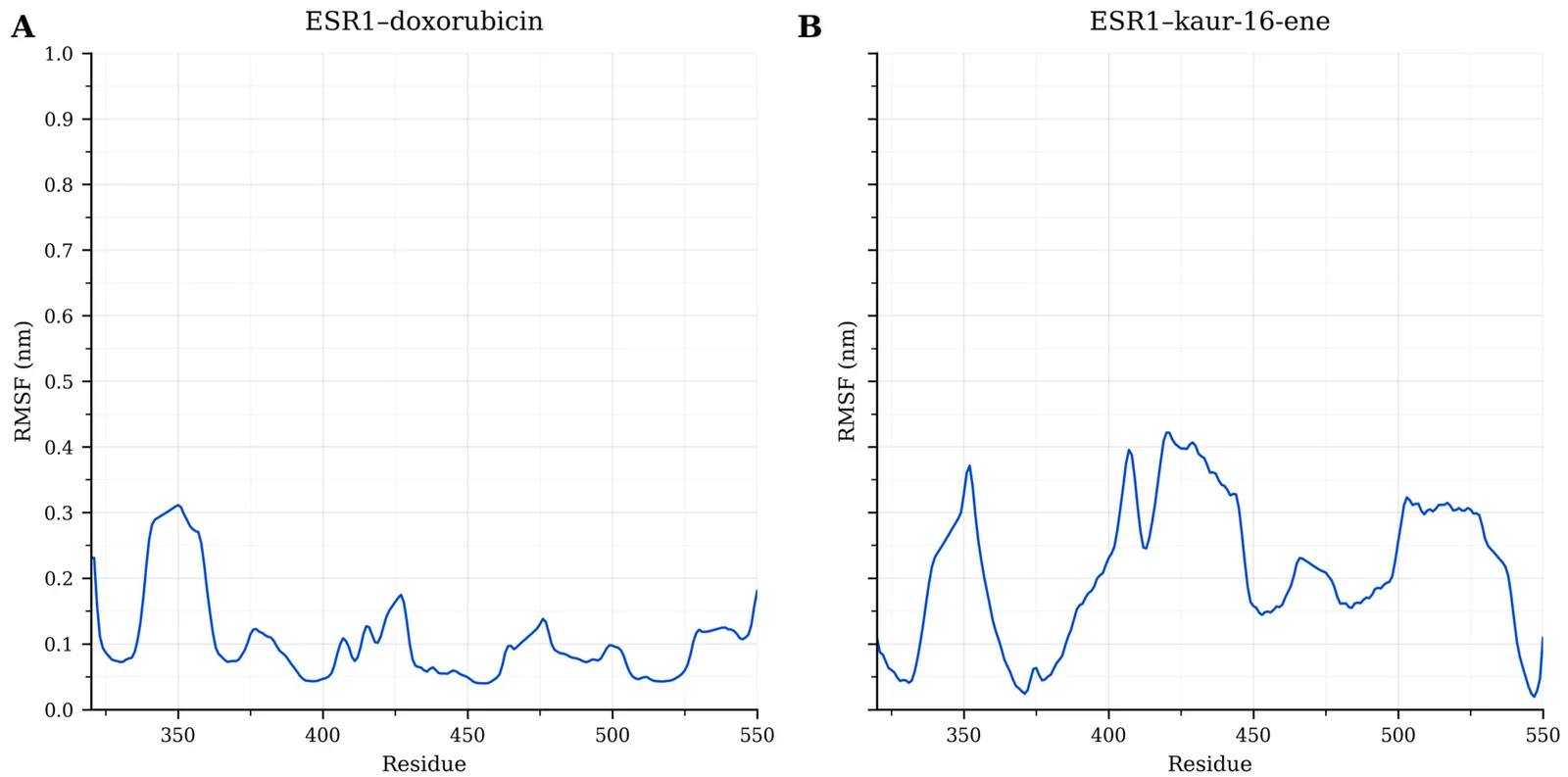
Root mean square fluctuation (RMSF) profiles of *ESR1* residues during 50 ns molecular dynamics simulation. (**A**) *ESR1*–doxorubicin reference complex. (**B**) *ESR1*–kaur-16-ene complex. RMSF values indicate residue-level flexibility across the simulated trajectory.

Root mean square fluctuation (RMSF) profiles are shown in Figure 11. In the *ESR1*–doxorubicin reference complex, most residues showed RMSF values below 0.15 nm, with higher fluctuations observed mainly in the Ser329–Ala350, Met388–Phe425, and Trp460–Leu470 regions. In the *ESR1*–kaur-16-ene complex, RMSF values were generally higher, with more pronounced fluctuations in the Ser329–Ala350, Leu391–Ala430, and Leu495–Arg515 regions. Thus, the RMSF analysis suggests that the kaur-16-ene-bound *ESR1* structure retained localized residue flexibility, particularly around selected loop or ligand-proximal regions, rather than uniform rigidity across the protein.

**Figure 12:**
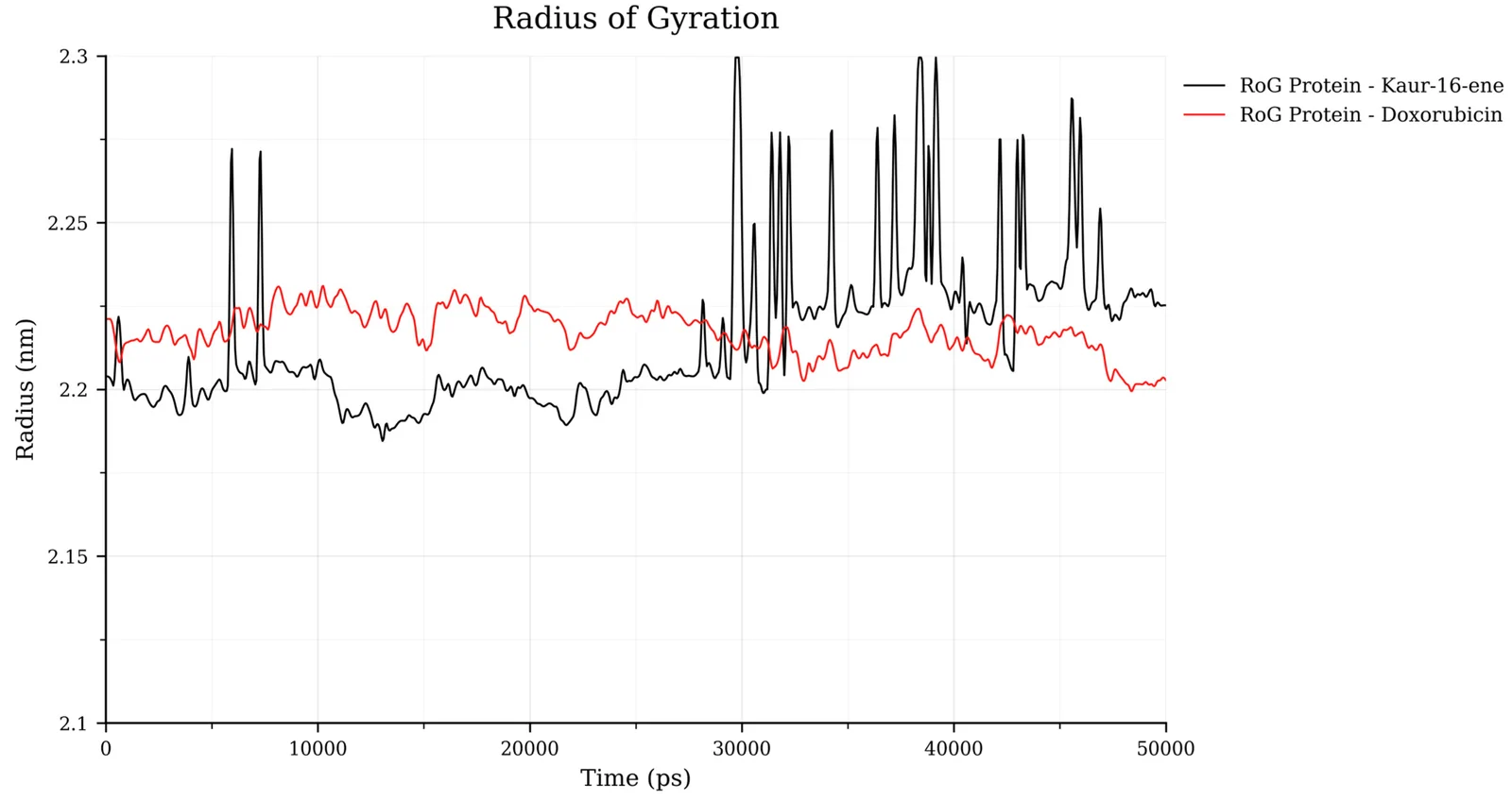
Radius of gyration (RoG) profiles of *ESR1* complexes during 50 ns molecular dynamics simulation for kaur-16-ene and doxorubicin reference compound. RoG values indicate changes in overall compactness of the protein–ligand complexes during the simulated trajectory.

Radius of gyration (RoG) profiles are shown in Figure 12. Both systems remained within a narrow range of approximately 2.19–2.24 nm throughout the simulation. In the *ESR1*–kaur-16-ene complex, the RoG value was slightly lower during the early phase and gradually approached the range observed for the doxorubicin reference system. In the *ESR1*–doxorubicin complex, the RoG values remained relatively constant over the simulation period. The comparable RoG profiles indicate that both *ESR1* complexes maintained similar overall compactness during the 50 ns trajectory.

Across the 50 ns simulation, the *ESR1*–kaur-16-ene complex demonstrated lower RMSD fluctuation than the *ESR1*–doxorubicin reference complex, while both systems maintained comparable RoG values. These results provide trajectory-based computational support for the short-timescale conformational stability of the *ESR1*–kaur-16-ene complex during the simulated timeframe. Together with the docking results, the molecular dynamics profiles support the prioritization of kaur-16-ene for future compound-level and target-focused validation.

## 3. Discussion

The present study showed that four Indonesian accessions of *A. compactum* exhibited antiproliferative activity across the three tested tumor cell models, although the magnitude of the response differed among accessions, extraction solvents, and cell models as shown by the IC_50_ profiles in Figure 1. A consistent feature of the in vitro assay was the lower IC_50_ values of several ethyl acetate extracts relative to the corresponding ethanol extracts, particularly in MCF-7 and MCM-B2 cells (Figure 1A,B). This pattern is consistent with the metabolite profiling data, which showed chemical variation among the extracts and identified terpenoids as the largest metabolite class in the dataset. In an Indonesian study on *A. compactum*, extraction conditions optimized for terpenoid recovery were also associated with cytotoxic activity, supporting the relevance of this species as a source of bioactive metabolites [23]. These results align with recent reports emphasizing that solvent polarity and extraction parameters significantly dictate the recovery of bioactive fractions in the genus *Amomum*, particularly for less-polar constituents [25]. The lower IC_50_ values observed for several ethyl acetate extracts suggest that compounds contributing to the antiproliferative effect were recovered more efficiently in this solvent system. This interpretation is chemically plausible because cardamom-associated fractions are enriched in less polar constituents, including mono- and sesquiterpenoid compounds. In the broader cardamom literature, cardamom has been described as a source of phytochemicals with potential relevance to cancer prevention and therapy, and cardamom essential oil has also shown cytotoxic activity against a breast cancer cell line, supporting the relevance of terpenoid-rich fractions as biologically active extracts [44,45]. Recent work on Java cardamom essential oil further supports the biological relevance of A. compactum fruit-derived volatile fractions, including antibacterial and angiotensin I-converting enzyme inhibitory activities [30]. At the same time, the extract-level response may reflect the combined contribution of multiple metabolite classes, because non-terpenoid constituents such as flavonoids, phenolic acids, phenylpropanoids, tannin-related phenolics, and fatty acids may also contribute individually or synergistically to the observed antiproliferative effects.

Another notable result was the accession-associated pattern of activity. The most active accession was not the same in all three cell models, indicating that the extracts did not behave uniformly across the tested models. For example, Ciamis ethyl acetate showed the lowest IC_50_ value in MCF-7 cells, Sukabumi ethyl acetate showed the lowest IC_50_ value in MCM-B2 cells, whereas Bogor white ethanol and Bogor red ethyl acetate showed stronger responses in MCA-B1 cells (Figure 1A–C). From a plant-science perspective, these findings indicate that the tested accessions differed in both extract-level activity and relative secondary-metabolite profiles. Such differences may be influenced by genetic, environmental, agronomic, or postharvest factors; however, the present study did not quantify individual metabolites or experimentally distinguish the contribution of these factors. In cardamom accessions from southern India, substantial variation in essential oil yield and composition was documented among accessions, supporting the interpretation that chemical diversity among plant materials can influence biological performance [46]. The chemometric analysis further supports the presence of chemical differentiation among the tested accession–solvent combinations. The PCA score plot showed separation among samples according to their LC-MS/MS profiles, and the loading plot indicated that this separation was driven by multiple metabolites rather than by a single dominant feature. Because Dim1 and Dim2 together explained 50.8% of the total variance, the PCA captures the main two-dimensional trend in the dataset and highlights the dominant accession–solvent separation pattern. The HCA heatmap supported this pattern by resolving the metabolites into distinct clusters and showing differences in their distribution among the extracts. Taken together, these results indicate that the ethanol and ethyl acetate extracts were chemically differentiated, and these profile differences were associated with, rather than demonstrated to cause, the observed differences in IC_50_-based antiproliferative responses.

The variation among cell models also deserves attention. Some accessions showed relatively strong activity in one model but weaker activity in another, suggesting that the observed effects depend, at least in part, on the cellular background of the test system. Responses to plant-derived agents in breast cancer are shaped by tumor biology, signaling context, and treatment-response mechanisms, and the present results are consistent with that broader pattern [47]. The tested panel included MCF-7 human breast cancer cells, MCM-B2 canine mammary tumor-derived cells, and MCA-B1 canine oral acanthomatous epulis-derived epithelial tumor cells; therefore, the observed response pattern is more accurately discussed as cell-model-dependent extract responsiveness rather than uniform breast-cancer-cell-line activity. MCF-7 is an estrogen receptor-positive human breast cancer model, and its response may be influenced by estrogen-associated survival and proliferation pathways [41]. In contrast, MCM-B2 is derived from a canine mammary mixed tumor, whereas MCA-B1 is derived from canine oral acanthomatous epulis and therefore differs from MCF-7 and MCM-B2 in both species origin and tissue origin [42,43]. These differences may affect basal proliferation rate, membrane permeability, stress-response capacity, metabolic state, and dependence on specific signaling pathways. In this respect, the antiproliferative effect of *A. compactum* extracts appears to be cell-model-dependent rather than uniform across the tested models. This interpretation is consistent with evidence that cancer cell models can display heterogeneous phenotypes and differential drug sensitivity, even within breast cancer cell-line systems [48]. Doxorubicin was included as an assay reference control, and its IC_50_ values across the tested cell models are shown in Figure 2. However, because doxorubicin is a purified reference compound and the cardamom extracts are chemically complex mixtures, these values should not be interpreted as direct potency equivalence between doxorubicin and crude extracts.

The LC-MS/MS analysis provided a chemical framework for these biological differences. A total of 36 metabolites were putatively annotated, and terpenoid-related compounds represented the largest chemical group. Several metabolites were detected repeatedly across accessions and solvent systems, whereas others showed more restricted occurrence patterns. This combination of shared and variable metabolites suggests that *A. compactum* contains both a recurring phytochemical background and accession-associated differences in relative metabolite distribution. In the earlier Indonesian study on *A. compactum*, terpenoid-rich extracts were likewise linked with cytotoxic activity, which is consistent with the prominence of terpenoids in the present dataset [23]. Overall, the secondary-metabolite profiling results identify accession- and solvent-associated chemical patterns that may be related to differences in extract-level antiproliferative responses.

The inclusion of Vero cells further allowed relative selectivity to be estimated using the Vero IC_50_ values shown in Figure 1D and the SI values summarized in Table 1. Several extracts showed comparatively higher SI values, particularly Ciamis ethyl acetate against MCF-7 and MCM-B2 cells and Sukabumi ethyl acetate against MCM-B2 cells (Table 1), suggesting stronger relative growth inhibition in those tumor cell models than in Vero cells. Because SI is calculated as the ratio of IC_50_ in non-tumor-derived cells to IC_50_ in tumor cells, higher SI values indicate greater relative selectivity [49,50]. However, SI values should be interpreted as extract-level relative selectivity indicators rather than proof of therapeutic selectivity, because Vero cells are a non-tumor-derived reference model and not a normal breast epithelial counterpart.

Because terpenoids were the dominant class in the present dataset, they were selected for subsequent bioinformatics analysis. This selection served as a terpenoid-focused compound-prioritization strategy while acknowledging that other metabolite classes may also contribute to extract-level activity. The network analysis identified 59 overlapping genes between predicted terpenoid targets and breast cancer-related genes, and the PPI analysis further narrowed these to ten hub genes, including *AKT1*, *HIF1A*, *CCND1*, *EGFR*, *BCL2*, *ESR1*, *GAPDH*, *JUN*, *SCN5A*, and *MTOR*. Several of these genes are closely related to cell-cycle control, survival signaling, endocrine response, and treatment resistance in breast cancer. AKT/mTOR signaling has been associated with resistance to endocrine therapy and CDK4/6 inhibition in metastatic breast cancer, while endocrine resistance in hormone receptor-positive disease is strongly linked to ESR-associated signaling and PI3K/AKT/mTOR pathway activity [51,52,53]. Recent reviews also emphasize that endocrine resistance is usually mediated by interconnected adaptive networks involving *ESR1* alterations, receptor tyrosine kinase signaling, and PI3K/AKT/mTOR pathway activation rather than by a single pathway alone [54,55]. These reports support the biological plausibility of the hub-gene profile identified in the present study. The enrichment analysis further supports this interpretation. The overrepresentation of pathways related to PI3K-Akt, HIF-1, FoxO, *EGFR* tyrosine kinase inhibitor resistance, and endocrine resistance indicates that the prioritized terpenoid-associated targets are linked to signaling programs involved in tumor growth and treatment response. Thus, the enrichment profile provides a pathway-level rationale for focusing subsequent docking and molecular dynamics analyses on selected breast cancer-related hub proteins.

Among the 15 terpenoid compounds examined, kaur-16-ene emerged as the most notable candidate in the docking analysis. It showed more favorable predicted binding than doxorubicin for *ESR1*, *EGFR*, *SCN5A*, and *CCND1*, while also retaining strong predicted interactions with several other targets. Within the applied docking workflow, this comparison was used to contextualize predicted ligand–protein interaction scores relative to the reference compound used in the in vitro assay. However, because doxorubicin is not a target-specific ligand for all selected hub proteins, this comparison should be interpreted only as a computational reference under the same docking protocol and not as evidence of superior pharmacological potency. Within the terpenoid set detected in *A. compactum*, kaur-16-ene was the ligand most consistently prioritized by the docking analysis. Kaur-16-ene belongs to the ent-kaurane diterpenoid class, which has been increasingly recognized in the recent literature for its potential antitumor properties and ability to modulate survival pathways in in vitro cancer models [29,56]. The interaction analysis showed that the binding mode of kaur-16-ene differed from that of doxorubicin. In the selected complexes, kaur-16-ene was stabilized mainly through hydrophobic contacts rather than hydrogen bonding. Given the structure of the ligand, this interaction pattern is chemically reasonable and is consistent with the nonpolar hydrocarbon nature of kaur-16-ene. The docking results therefore suggest that hydrophobic packing contributed substantially to the predicted placement of kaur-16-ene within the binding pockets of *ESR1*, *EGFR*, *SCN5A*, and *CCND1*. These interaction patterns support the prioritization of kaur-16-ene for subsequent compound-level and target-focused evaluation.

The molecular dynamics results provide additional support for the *ESR1*–kaur-16-ene complex. Compared with the *ESR1*–doxorubicin reference system, the kaur-16-ene complex showed lower RMSD fluctuation over the 50 ns simulation, whereas the RoG values remained within a narrow range in both systems. The RMSF profile indicated increased flexibility in several residue regions in the kaur-16-ene system, but this was not accompanied by major structural drift. Taken together, these results support short-timescale conformational stability of the *ESR1*–kaur-16-ene complex under the simulation conditions used here and suggest that the ligand can be theoretically accommodated without substantial destabilization of the receptor conformation. Given the established role of *ESR1* in hormone-responsive breast cancer, this result is mechanistically relevant to the breast cancer-related target-prioritization workflow [51]. Additional computational refinement using longer simulation trajectories and binding free-energy approaches, such as MM-PBSA or MM-GBSA, would further strengthen the evaluation of complex stability and ligand–receptor interaction energetics. Although *SCN5A* is classically associated with voltage-gated sodium channel function, its inclusion in the predicted network is biologically plausible because voltage-gated sodium channels, including NaV1.5 encoded by *SCN5A*, have been implicated in cancer-cell proliferation, migration, invasion, and metastatic behavior [57,58]. Compared with *ESR1*, *EGFR*, *CCND1*, and AKT/*MTOR*-related signaling, *SCN5A* is best positioned as an exploratory ion-channel-associated node in the present analysis.

This study also has several limitations. First, the metabolites were annotated at the extract level, and the contribution of individual compounds to the observed antiproliferative effect was not tested directly using isolated molecules. Moreover, metabolite annotation was putative because authentic standards were not used for compound confirmation. The LC-MS/MS analysis also did not include calibration curves or targeted quantification; therefore, the metabolite data support relative profiling and chemometric comparison rather than absolute concentration-based chemical characterization. Second, although IC_50_ values were determined from concentration–response assays, the biological activity remains extract-level and does not identify which individual metabolites are responsible for the observed effects. Furthermore, the observed IC_50_ values for the crude extracts fall within the milligram per milliliter (mg mL^−1^) range, in contrast to the microgram-level (µg mL^−1^) potency exhibited by the reference drug doxorubicin. This milligram-scale efficacy represents an inherent limitation of unpurified, multi-component botanical extracts, where non-cytotoxic bulk metabolites dilute bioactive constituents. Consequently, these baseline values serve primarily as comparative metrics for accession screening rather than indicators of immediate clinical potency. Third, Vero cells were included to estimate relative selectivity; however, Vero cells are not normal breast epithelial cells, and SI values therefore cannot establish breast-tissue-specific safety or therapeutic selectivity. Fourth, the target prediction, docking, and molecular dynamics analyses provide supportive mechanistic hypotheses, but they do not replace biochemical or cellular target validation. The predicted ADMET results also indicate that kaur-16-ene has high lipophilicity, low predicted gastrointestinal absorption, and possible CYP inhibition, which may constrain its pharmacokinetic suitability despite favorable docking scores. Thus, kaur-16-ene should be regarded as a computationally prioritized candidate for validation rather than as a confirmed lead compound. In the broader context of herbal medicine in breast cancer, phytochemical screening and in silico prioritization still require compound-level and target-level validation before stronger mechanistic conclusions can be made [47].

Overall, the present results support three main points. First, Indonesian accessions of *A. compactum* showed differences in relative secondary-metabolite profiles and IC_50_-based antiproliferative response. Second, the lower IC_50_ values of several ethyl acetate extracts, particularly in MCF-7 and MCM-B2 cells, are consistent with the recovery of relatively less-polar terpenoid-associated fractions, although non-terpenoid metabolites may also contribute to the extract-level responses. Third, the combined in vitro, secondary-metabolite profiling, network, docking, and molecular dynamics analyses prioritize kaur-16-ene as a candidate compound for further investigation. The inclusion of Vero cells and SI calculation strengthened the preliminary biological interpretation by adding a relative selectivity component; however, this remains an early extract-level assessment. The most direct next step would be to isolate kaur-16-ene and related terpenoids, evaluate them individually in dose–response assays, compare toxicity against normal breast epithelial and other non-tumorigenic cell models, and validate the predicted targets experimentally in appropriate human breast cancer and comparative tumor-derived cell models [34,47,56]. Future studies should also incorporate bioassay-guided fractionation, authentic-standard confirmation and targeted quantification of key metabolites, non-terpenoid target prediction, apoptosis and cell-cycle assays, longer molecular dynamics simulations, and binding free-energy estimation to establish stronger mechanistic evidence.

## 4. Materials and Methods

### 4.1. Plant Materials and Authentication

This study used four local cardamom (*A. compactum*) mature fruit accessions collected from West Java, Indonesia, in May 2020. Two accessions originated from Bogor and were distinguished based on pod color, namely Bogor white cardamom (BOW/Bogor-1) and Bogor red cardamom (BOR/Bogor-2). The other two accessions were obtained from Sukabumi (SI) and Ciamis (CI). The collection sites were located in Bogor (6°43′30.4″ S; 106°41′40.6″ E; 1267 m asl), Sukabumi (7°17′38.5″ S; 106°50′19.6″ E; 262 m asl), and Ciamis (7°19′03.0″ S; 108°19′56.9″ E; 246 m asl). Mature fruits were harvested from IPB University partner farmers at physiological maturity and used as the plant material for extraction and metabolomic analysis. The plant materials were authenticated and deposited at the Tropical Biopharmaca Research Center, IPB University, Bogor, Indonesia. The voucher specimen numbers were BMK0472052020 for Bogor-1, BMK0473052020 for Bogor-2, BMK0474052020 for Sukabumi, and BMK0475052020 for Ciamis. The species name was retained as *A. compactum* to maintain consistency with the authenticated voucher records and the Java cardamom literature, while current taxonomic databases list *A. compactum* Sol. ex Maton as a synonym of *Wurfbainia compacta* (Sol. ex Maton) Škorničk. & A.D.Poulsen [14,15,59].

### 4.2. Cardamom Sample Extraction

Cardamom fruits from each accession were oven-dried at 45 °C for 2 days until the moisture content was below 10%. The dried fruits were stored in sealed polyethylene bags at room temperature under dry conditions before extraction. The dried samples were then ground into powder and sieved through an 80-mesh sieve to obtain a uniform particle size. Extraction was performed separately using 80% ethanol and ethyl acetate as solvents, following a sonication-assisted method adapted from previous research [60] with minor modifications. The use of two solvents was intended to obtain complementary metabolite fractions because solvent polarity strongly influences phytochemical recovery from plant matrices; therefore, 80% ethanol and ethyl acetate were used to broaden the range of extracted polar, semi-polar, and relatively less-polar constituents [61,62]. Briefly, 10 g of powdered sample was extracted twice with 50 mL of solvent, corresponding to a sample-to-solvent ratio of 1:5 (*w*/*v*) for each extraction cycle. The mixtures were placed in extraction tubes and sonicated using a Decon F5 Major ultrasonic bath sonicator (Decon Ultrasonics Ltd., Sussex, England, UK) for 30 min under dark conditions. The extraction was performed at room temperature. The sample temperature during sonication was not continuously monitored; therefore, no specific sonication temperature range is reported. After extraction, the homogenates were centrifuged at 10,000× *g* and 4 °C for 15 min to separate the supernatant. The collected supernatants were then concentrated using a rotary vacuum evaporator at 40 °C under reduced pressure until the extraction solvent was removed. The resulting extracts were stored at −4 °C in light-protected containers until further use in antiproliferation assays and LC-MS/MS analysis.

### 4.3. LC-MS/MS Analysis

Metabolomic profiling of the cardamom extracts was performed using an untargeted liquid chromatography–tandem mass spectrometry (LC-MS/MS) approach, with instrumental conditions adapted from a previously reported metabolomics method [63]. Ethanol (80%) and ethyl acetate extracts of cardamom, each prepared at a concentration of 0.2 g mL^−1^, were analyzed using a UHPLC Vanquish system coupled to a Q Exactive Plus Orbitrap high-resolution tandem mass spectrometer (Thermo Scientific, Waltham, MA, USA). Prior to analysis, sample stocks were dissolved in 5 mL of LC-MS-grade methanol (Merck, Darmstadt, Germany), sonicated, and filtered through a 0.2 μm syringe filter membrane (SY25TF PTFE mdi); 5 μL of each filtrate was injected into the LC system. 

Chromatographic separation was carried out on an Accucore™ Phenyl-Hexyl column (100 × 2.1 mm, 2.6 µm; Thermo Scientific, Waltham, MA, USA) at a flow rate of 0.2 mL min^−1^ using a mobile phase consisting of LC-MS-grade water (Merck, Darmstadt, Germany) with 0.1% formic acid (≥98%; Merck, Darmstadt, Germany) as solvent A and LC-MS-grade acetonitrile (≥99.9%; Merck, Darmstadt, Germany) with 0.1% formic acid as solvent B, under a 15 min gradient program (0 min, 30% B; 0–1.7 min, 40% B; 1.7–6.9 min, 75% B; 6.9–7.8 min, 100% B; 7.8–8.2 min, 30% B; and 8.2–15 min, 30% B). Mass spectrometric detection was performed using an electrospray ionization (ESI) source in both positive- and negative-ion modes with a scan range of *m*/*z* 100–1500, a resolving power of 70,000 FWHM, a spray voltage of 3.8 kV, and a capillary temperature of 320 °C, while MS/MS fragmentation was conducted using collision energies of 18, 35, and 53 eV. 

Putative metabolite annotation was performed in accordance with the Metabolomics Standards Initiative (MSI) guidelines and was reported as MSI level 2 unless otherwise indicated [64]. Compound annotation was based on accurate mass, retention time, and MS/MS fragmentation patterns obtained from the LC-MS/MS analysis and software-assisted spectral matching. Because authentic reference standards were not analyzed, the metabolite assignments were considered putative rather than definitive structural identifications. The raw data were processed using Compound Discoverer software version 2.1.0.401 (Thermo Scientific, Waltham, MA, USA) for peak detection, alignment, and metabolite annotation. Peak-area data generated from the processed LC-MS/MS features were compiled for comparative chemometric analysis across accession–solvent combinations. The curated annotation output included metabolite name, chemical class, retention time, molecular formula, molecular weight, precursor ion, product ions, MSI confidence level, and accession–solvent detection profile.

### 4.4. Antiproliferation Assay on MCF-7, MCM-B2, MCA-B1, and Vero Cell Models

The antiproliferative activity of the cardamom extracts was evaluated in MCF-7 human breast cancer cells, MCM-B2 canine mammary tumor-derived cells, and MCA-B1 canine oral acanthomatous epulis-derived epithelial tumor cells using a trypan blue exclusion assay to measure cell viability, following previously reported methods with minor modifications [65,66]. Vero cells were included as a non-tumor-derived reference cell model to evaluate the relative selectivity of the extracts. MCF-7 was used as a human breast cancer cell model, whereas MCM-B2 and MCA-B1 were included as comparative tumor-derived cell models. Dulbecco’s Modified Eagle Medium (DMEM) containing phenol red was prepared by adding 0.1% gentamicin, 0.1% fungizone, and 10% fetal bovine serum (FBS). The cardamom extracts were prepared using dimethyl sulfoxide (DMSO) and evaluated at serial concentrations of 0.03125, 0.0625, 0.125, 0.25, 0.5, 1, 2, 4, and 8 mg mL^−1^ to generate concentration–response data for IC_50_ determination. Doxorubicin was used as the positive control and evaluated at concentrations of 1.5625, 3.125, 6.25, 12.5, 25, 50, 100, and 200 µg mL^−1^. The preserved MCF-7, MCM-B2, MCA-B1, and Vero cell suspensions were thawed and homogenized using a vortex mixer for 10 min. The assay was designed as a concentration–response experiment to determine IC_50_ values and to assess the relative selectivity of the extracts toward tumor-derived cells compared with Vero cells.

Cells were seeded in 24-well tissue culture plates containing DMEM growth medium and treated with the different cardamom extracts or doxorubicin at the specified serial concentrations. Cells without extract treatment were used as the negative control, whereas doxorubicin (Sigma-Aldrich, St. Louis, MO, USA) was used as the positive control. Because the extracts and doxorubicin were tested at different concentrations and chemical complexities, doxorubicin was used only as an assay reference control and not for direct potency equivalence with the crude extracts. Each well contained 900 μL of DMEM, 50 μL of cell suspension, and 50 μL of extract or positive-control solution. The experiment was conducted in triplicate, and the treated cells were incubated at 37 °C in a 5% CO_2_ incubator. Cells were harvested when the control wells reached approximately 70% confluence, typically after three days of incubation. The cell suspension was homogenized by repeated aspiration and dispensing using a micropipette. A total of 80 μL of cell suspension was mixed with 20 μL of trypan blue dye (Sigma-Aldrich, St. Louis, MO, USA) to distinguish viable and non-viable cells. The mixture was loaded onto a Neubauer hemocytometer (Paul Marienfeld GmbH & Co. KG, Lauda-Königshofen, Germany), and cells were counted under a light microscope at 100× magnification. Cell growth and inhibition were calculated using the following formulas:

$$\mathrm{Growth}\;\mathrm{activity}\;(\%)=\frac{\sum \mathrm{average}\;\mathrm{treatment}\;\mathrm{cells}}{\sum \mathrm{average}\;\mathrm{negative}\;\mathrm{control}\;\mathrm{cells}}\times 100$$

Inhibition activity (%)=100−Growth activity (%)

The percentage of inhibition was used as a quantitative indicator of antiproliferative activity across the tested tumor cell models. The inhibition values obtained across the tested concentrations were used to calculate IC_50_ values by nonlinear regression analysis. Extract IC_50_ values were expressed in mg mL^−1^, whereas doxorubicin IC_50_ values were expressed in µg mL^−1^.

To evaluate the relative selectivity of the extracts, SI was calculated by dividing the IC_50_ value in Vero cells by the IC_50_ value in each tumor cell model, as commonly applied in cytotoxicity selectivity assessment [49,50]:

$$\mathrm{SI}=\frac{{\mathrm{IC}}_{50,\;\mathrm{Vero}}}{{\mathrm{IC}}_{50,\;\mathrm{tumor}}}$$

Higher SI values indicate greater relative selectivity toward tumor-derived cells compared with Vero cells.

### 4.5. Data Analysis

All in vitro experiments were set up using a randomized complete block design with cardamom accession as the single factor, and all measurements were performed in triplicate. Percentage inhibition data obtained from serial concentrations were used to generate concentration–response curves for each extract and doxorubicin treatment. IC_50_ values were estimated by nonlinear regression analysis using GraphPad Prism version 11.0.1 (GraphPad Software, Boston, MA, USA). Extract IC_50_ values were expressed in mg mL^−1^, whereas doxorubicin IC_50_ values were expressed in µg mL^−1^. 

For extract IC_50_ values, statistical differences were analyzed separately for each cell model using two-way analysis of variance (ANOVA), with cardamom accession and extraction solvent as the two fixed factors. The accession × solvent interaction was also evaluated to determine whether the effect of extraction solvent differed among accessions. When significant effects or interactions were detected, multiple comparisons were performed using Tukey’s post hoc test. Differences were considered statistically significant at *p* < 0.05. Doxorubicin IC_50_ values were analyzed separately because doxorubicin was used as a reference compound and was not part of the accession–solvent factorial design. 

To evaluate variation in relative secondary-metabolite profiles across accession–solvent combinations, multivariate analyses, specifically principal component analysis (PCA) and hierarchical cluster analysis (HCA), were performed using the R software (version 4.5.2) environment within RStudio (version 2025.09.0+387) (Posit Software, PBC, Boston, MA, USA). Before the analysis, the peak areas of putatively annotated metabolites were compiled into a data matrix. Any missing values, corresponding to undetected metabolites in specific accession–solvent combinations, were imputed using half of the dataset’s minimum positive value. The data then underwent log10 transformation and autoscaling before multivariate analysis to normalize intensity variations. Finally, the PCA models and biplots were generated using the FactoMineR and factoextra packages (FactoMineR version 2.14, factoextra version 2.0.0), while the HCA and heatmap visualizations were created using the pheatmap package (version 1.0.13). Because the LC-MS/MS workflow was untargeted and did not use calibration curves or authentic standards for quantification, peak-area data were interpreted as relative abundance patterns rather than absolute concentrations.

### 4.6. Target Identification and Network Analysis

#### 4.6.1. Prediction of Target Genes

The terpenoid-related compounds putatively annotated from the LC-MS/MS results were selected for further bioinformatics analysis as a focused compound-prioritization strategy. SwissTargetPrediction was used to predict potential target genes associated with the selected compounds using *Homo sapiens* as the target organism when applicable [67]. Breast cancer-related genes were retrieved from OMIM [68], UniProt [69], GeneCards [70], and TCGA through the Genomic Data Commons data portal. The search was performed using the keywords “breast cancer” and “TCGA-BRCA”. Genes unrelated to breast cancer were excluded to minimize ambiguity in the analysis. The resulting target list was treated as a database-derived candidate target set for downstream network analysis.

#### 4.6.2. Venn Diagram Construction

The intersection between the predicted terpenoid compound targets and the retrieved breast cancer-related genes was identified using a Venn diagram. Overlapping genes were considered candidate breast cancer-associated terpenoid targets and used for subsequent network analysis. This intersection step was used to prioritize genes shared between the compound-predicted target set and the breast-cancer-associated gene set.

#### 4.6.3. Protein–Protein Interaction Network

Protein–protein interaction (PPI) analysis was performed using STRING, with the organism set to *Homo sapiens* and a minimum confidence score of 0.7 to obtain high-confidence interactions [71]. The resulting network was exported to Cytoscape version 3.10.4 for visualization and topological analysis [72]. Clustering analysis was performed using the Cytocluster plugin (version 2.1.0 ) with the ClusterONE algorithm, whereas key target genes were selected based on degree centrality. The ten genes with the highest degree values were selected as network-derived hub genes for molecular docking analysis. Hub-gene ranking was based on degree centrality within the constructed PPI network.

#### 4.6.4. Enrichment Analysis

Gene Ontology (GO) and Kyoto Encyclopedia of Genes and Genomes (KEGG) enrichment analyses were performed using the ShinyGO platform to computationally interpret the biological functions, cellular processes, and molecular pathways associated with the overlapping target genes. The overlapping target-gene list was used as the input dataset, and enriched GO terms and KEGG pathways were ranked according to statistical significance. The significance threshold was set at *p* < 0.05 [73,74,75].

### 4.7. Ligand and Protein Preparation

#### 4.7.1. Ligand Preparation and ADMET Prediction

The terpenoid compounds putatively annotated from the LC-MS/MS analysis were used as ligands. The three-dimensional structures of the compounds were obtained from the PubChem database [76] and saved in simple data format (SDF). Doxorubicin was used as the reference ligand because it was used as the positive control in the in vitro assay. Ligand suitability as drug candidates was evaluated using SwissADME based on Lipinski’s rule of five [77]. Predicted absorption, distribution, metabolism, excretion, and toxicity (ADMET) properties were also compiled to support pharmacokinetic interpretation. Gastrointestinal absorption, blood–brain barrier permeability, cytochrome P450 inhibition, and skin permeability were obtained from SwissADME, whereas acute oral toxicity parameters, including predicted LD_50_ and toxicity class, were predicted using ProTox-II [78]. Energy minimization of the ligand structures was performed using the Open Babel tool in PyRx version 0.8 [79]. The prepared ligands were then saved in .pdbqt format.

#### 4.7.2. Protein Preparation

Ten target genes identified from the PPI analysis were used as receptors. Protein structures of the target genes, namely *GAPDH* (PDB ID: 1U8F), *ESR1* (PDB ID: 9BU1), *AKT1* (PDB ID: 6HHJ), *EGFR* (PDB ID: 8HV2), *CCND1* (PDB ID: 2W96), *SCN5A* (PDB ID: 7DTC), *BCL2* (PDB ID: 8HTS), *JUN* (PDB ID: 1JNM), *HIF1A* (PDB ID: 3KCX), and *MTOR* (PDB ID: 5H64), were obtained from the RCSB Protein Data Bank [80]. Protein preparation was performed by removing water molecules, co-crystal ligands, and unnecessary residues using UCSF ChimeraX version 1.11 [81]. Polar hydrogen atoms and partial charges were added using AutoDock Tools version 1.5.7. The processed proteins were saved in .pdbqt format [82].

### 4.8. Molecular Docking

Molecular docking was performed using AutoDock Vina in PyRx version 0.8 [83,84,85]. The x, y, and z coordinates of the grid box were determined using the PrankWeb platform by selecting the first and highest-ranking predicted pocket as the active site of each protein [86] (Table S2). Docking results were analyzed based on predicted binding affinity (binding energy, expressed in kcal mol^−1^) and interaction patterns between the ligands and amino acid residues of the target proteins. Molecular interactions were visualized using PyMOL Molecular Graphics System version 3.1.6.1 and BIOVIA Discovery Studio Visualizer version 25.1.0.2484. Docking scores were used to prioritize ligand–protein pairs for interaction analysis and molecular dynamics simulation.

### 4.9. Molecular Dynamics Simulation

Molecular dynamics (MD) simulations were performed using GROMACS 2024.4 to evaluate the short-timescale conformational behavior of the protein–ligand complexes showing the best interactions in the molecular docking analysis [87]. Protein and ligand preparation involved re-separating the docked ligands using UCSF ChimeraX and exporting them in .pdb format. The ligand files were then converted to MOL2 format using Open Babel and parameterized using SwissParam. System topology was constructed using the Chemistry at Harvard Macromolecular Mechanics 36 (CHARMM36) force field [88,89]. The complexes were solvated in a cubic TIP3P water box with a minimum distance of 1.0 nm from the box edge. Na^+^ and Cl^−^ ions were added to neutralize the system charge [90]. Energy minimization was performed to eliminate steric clashes and stabilize the initial structure. The equilibration process was conducted in two stages: constant volume and temperature (NVT) equilibration at 300 K, followed by constant pressure and temperature (NPT) equilibration at 1 atm. Production MD simulations were performed for 50 ns with a 2 fs time step under periodic boundary conditions. The resulting trajectories were reanalyzed through recentering and rewrapping. Structural stability and dynamics of the complexes were evaluated using root mean square deviation (RMSD), root mean square fluctuation (RMSF), and radius of gyration (RoG). Visualization was performed using XMGrace. The MD simulation was used to assess trajectory-based conformational behavior of selected complexes over the simulated timeframe.

## 5. Conclusions

This study showed that Indonesian *A. compactum* accessions exhibit distinct metabolite compositions and accession- and solvent-dependent extract-level antiproliferative responses across the tested tumor cell models in vitro, as indicated by IC_50_-based concentration–response analysis. Ethyl acetate extracts generally showed stronger antiproliferative effects than ethanol extracts, particularly against MCF-7 and MCM-B2 cells, suggesting that relatively less-polar constituents may be associated with the observed extract-level responses. The inclusion of Vero cells enabled preliminary estimation of relative selectivity, with selected extracts showing higher selectivity indices toward MCF-7 and MCM-B2 cells than toward MCA-B1 cells. Untargeted LC-MS/MS profiling putatively annotated 36 metabolites, with terpenoid-related compounds representing the predominant chemical group. Computational network analysis prioritized ten network-derived hub genes, while molecular docking and molecular dynamics analyses computationally prioritized kaur-16-ene as a terpenoid candidate, particularly through its predicted interaction with *ESR1* and other breast-cancer-associated network proteins, including *EGFR*, *SCN5A*, and *CCND1*. Together, these findings position kaur-16-ene as a computationally prioritized candidate for further compound-level and target-focused validation.

These findings support the potential of Indonesian *A. compactum* as a source of bioactive metabolites, particularly terpenoid-related compounds, and as valuable germplasm for further investigation. The results also define clear priorities for future validation, including authentic-standard confirmation and targeted quantification of key metabolites, bioassay-guided fractionation, metabolite isolation, isolated-compound dose–response assays, comparison with normal breast epithelial and other non-tumorigenic cell models, isolated-compound testing, apoptosis and cell-cycle analyses, experimental target validation, and ADMET evaluation. Because the present selectivity assessment was based on Vero cells, future studies should include tissue-relevant normal cell models to better evaluate safety and therapeutic selectivity. Furthermore, multilocation trials, phenotypic characterization, agronomic evaluation, and varietal-stability assessment are required before specific accessions can be recommended for varietal development or preclinical application.

## Figures and Tables

**Table 1:** Selectivity index (SI) of ethanol and ethyl acetate extracts from four Indonesian cardamom (*Amomum compactum*) accessions based on IC_50_ values in Vero and tumor-derived cell models.

Extraction Solvent	Cardamom Accession	Vero IC_50_ (mg/mL)	SI vs. MCF-7	SI vs. MCM-B2	SI vs. MCA-B1
Ethanol	Sukabumi	4.00	3.21	1.74	1.17
Ethanol	Bogor white	2.81	1.92	1.53	2.03
Ethanol	Ciamis	4.00	1.81	1.46	1.40
Ethanol	Bogor red	3.14	1.99	1.53	1.95
Ethyl acetate	Sukabumi	2.90	1.94	3.55	1.77
Ethyl acetate	Bogor white	2.76	2.63	2.69	1.64
Ethyl acetate	Ciamis	3.65	3.81	4.06	1.17
Ethyl acetate	Bogor red	3.43	2.76	1.73	2.35

Note: IC_50_, half-maximal inhibitory concentration; SI, selectivity index. SI was calculated as Vero IC_50_ divided by tumor-cell IC_50_. Higher SI values indicate greater relative selectivity toward tumor cells compared with non-tumor Vero cells. IC_50_ values were estimated from concentration–response analysis.

**Table 2:** Metabolites putatively annotated by LC-MS/MS in four Indonesian cardamom (*Amomum compactum*) accessions extracted with ethanol and ethyl acetate, including Metabolomics Standards Initiative (MSI) confidence levels and accession-solvent detection profiles.

No.	Metabolites	Chemical Class	RT (min)	Formula	MW (Da)	MSI Level	Precursor ion (*m*/*z*)	Product ion (*m*/*z*)	CI-EtOAc	CI-EtOH	BOR-EtOAc	BOR-EtOH	BOW-EtOAc	BOW-EtOH	SI-EtOAc	SI-EtOH
1	Norcamphor	Monoterpenoid	1.644	C_7_H_10_O	110.0730	2	111.0803	67.05, 81.07, 93.07	ND	ND	ND	ND	ND	✓	ND	✓
2	Salicylic acid	Phenolic acid	1.777	C_7_H_6_O_3_	138.0304	2	137.0231	93.03, 65.03	✓	✓	✓	ND	✓	✓	✓	✓
3	Gallic acid	Phenolic acid	1.788	C_7_H_6_O_5_	170.0204	2	169.0131	125.02, 107.01, 79.02	✓	ND	ND	ND	✓	ND	ND	ND
4	Catechin	Flavonoid	1.935	C_15_H_14_O_6_	290.0783	2	291.0854	139.04, 123.04, 109.03	ND	ND	ND	✓	ND	✓	ND	ND
5	Citral	Monoterpenoid	1.958	C_10_H_16_O	152.1195	2	153.1268	95.08, 109.08, 121.09	ND	✓	ND	ND	✓	✓	ND	✓
6	Quercetin-3β-D-glucoside	Flavonoid glycoside	1.969	C_21_H_20_O_12_	464.0945	2	465.1016	301.03, 179.03, 151.00	✓	ND	ND	ND	✓	✓	ND	✓
7	Ellagic acid	Tannin-related phenolic	2.020	C_14_H_6_O_8_	302.0058	2	300.9985	257.01, 229.01, 185.02	ND	ND	ND	ND	ND	✓	ND	✓
8	Thymol	Monoterpenoid phenol	2.178	C_10_H_14_O	150.1039	2	151.1112	135.08, 107.05, 91.05	✓	✓	✓	ND	✓	ND	✓	✓
9	(+)-Limonene	Monoterpene hydrocarbon	2.328	C_10_H_16_	136.1247	2	137.1320	95.08, 109.08, 81.07	✓	ND	✓	ND	✓	✓	✓	ND
10	10-Undecenoic acid	Fatty acid	2.377	C_11_H_20_O_2_	184.1458	2	185.1530	165.13, 137.13, 123.12	✓	ND	✓	✓	✓	✓	✓	✓
11	Pulegone	Monoterpenoid	2.510	C_10_H_16_O	152.1195	2	153.1268	81.07, 109.08, 135.12	✓	ND	✓	ND	ND	✓	✓	✓
12	Quercitrin	Flavonoid glycoside	2.552	C_21_H_20_O_11_	448.0996	2	449.1069	301.03, 151.00, 179.03	ND	ND	ND	✓	ND	ND	ND	ND
13	Dihydrosamidin	Coumarin	2.566	C_21_H_24_O_7_	388.1510	2	389.1583	245.08, 227.07, 175.04	ND	ND	ND	ND	ND	ND	ND	✓
14	p-Cymene	Monoterpene hydrocarbon	2.721	C_10_H_14_	134.1091	2	135.1162	119.08, 91.05, 105.07	✓	✓	✓	✓	✓	✓	✓	✓
15	Hyperoside	Flavonoid glycoside	2.740	C_21_H_20_O_12_	464.0945	2	465.1018	301.03, 271.02, 151.00	ND	ND	ND	✓	ND	ND	ND	ND
16	D-(+)-camphor	Monoterpenoid	2.779	C_10_H_16_O	152.1196	2	153.1268	81.07, 95.08, 109.08	✓	ND	✓	✓	ND	ND	✓	✓
17	Vanillin	Phenolic aldehyde	2.951	C_8_H_8_O_3_	152.0468	2	151.0389	123.04, 108.02, 93.03	ND	ND	✓	✓	ND	✓	✓	✓
18	Eugenol	Phenylpropanoid	3.279	C_10_H_12_O_2_	164.0834	2	165.0907	149.06, 131.05, 121.06	ND	✓	ND	✓	ND	ND	ND	ND
19	Carvone	Monoterpenoid	3.479	C_10_H_14_O	150.1039	2	151.1112	109.07, 123.08, 93.07	✓	✓	✓	✓	✓	✓	✓	ND
20	Decanoic acid	Fatty acid	3.648	C_10_H_20_O_2_	172.1459	2	173.1532	155.14, 127.11, 113.10	ND	✓	ND	ND	ND	✓	ND	ND
21	Quercetin	Flavonoid	3.697	C_15_H_10_O_7_	302.0419	2	301.0348	151.00, 179.03, 107.01	ND	ND	ND	✓	ND	✓	ND	✓
22	α-Pinene-2-oxide	Monoterpenoid	4.019	C_10_H_16_O	152.1195	2	153.1268	109.08, 95.08, 81.07	ND	✓	ND	ND	ND	ND	ND	ND
23	3-Hydroxybenzoic acid	Phenolic acid	4.284	C_7_H_6_O_3_	138.0304	2	137.0232	93.03, 65.03	ND	ND	ND	✓	ND	ND	ND	ND
24	(E)-4-Methoxycinnamic acid	Cinnamic acid derivative	4.513	C_10_H_10_O_3_	178.0625	2	179.0698	161.06, 133.07, 105.07	ND	✓	ND	ND	ND	ND	ND	ND
25	L-(-)-carvone	Monoterpenoid	4.672	C_10_H_14_O	150.1040	2	151.1112	109.07, 123.08, 93.07	ND	✓	ND	ND	ND	ND	ND	✓
26	4-Hydroxybenzoic acid	Phenolic acid	4.804	C_7_H_6_O_3_	138.0305	2	137.0232	93.03, 65.03	ND	✓	ND	✓	ND	ND	ND	ND
27	4-Isopropylphenol	Alkylphenol	5.107	C_9_H_12_O	136.0885	2	137.0957	121.06, 91.05, 77.04	ND	✓	✓	✓	✓	✓	ND	✓
28	1,8-Cineole	Monoterpenoid	6.253	C_10_H_18_O	154.1358	2	155.1424	93.07, 109.07, 121.09	✓	✓	✓	✓	✓	✓	✓	✓
29	(+)-Alantolactone	Sesquiterpene lactone	6.961	C_15_H_20_O_2_	232.1454	2	233.1527	187.15, 159.12, 105.07	ND	✓	ND	ND	ND	✓	ND	✓
30	(E,E)-α-farnesene	Sesquiterpene hydrocarbon	8.826	C_15_H_24_	204.1869	2	205.1941	121.09, 107.08, 93.07	✓	✓	✓	✓	✓	✓	✓	✓
31	(2E,6E)-farnesol	Sesquiterpenoid alcohol	8.997	C_15_H_26_O	222.1976	2	223.2049	161.13, 121.09, 107.08	✓	✓	✓	✓	✓	✓	✓	✓
32	Tiglic acid	Unsaturated short-chain fatty acid	9.001	C_5_H_8_O_2_	100.0523	2	101.0596	83.05, 55.05	✓	ND	ND	ND	✓	ND	✓	ND
33	Linolenic acid	Polyunsaturated fatty acid	9.418	C_18_H_30_O_2_	278.2233	2	277.22	95.05, 109.06, 123.08	✓	✓	✓	ND	ND	✓	✓	✓
34	Vitamin A	Vitamin/retinoid	10.271	C_20_H_30_O	286.2287	2	287.2360	269.23, 161.09, 145.09	✓	ND	✓	✓	✓	ND	✓	ND
35	Oleic acid	Monounsaturated fatty acid	10.638	C_18_H_34_O_2_	282.2550	2	283.2622	265.25, 245.23, 111.08	✓	✓	✓	✓	✓	✓	✓	✓
36	Kaur-16-ene	Diterpene hydrocarbon	10.678	C_20_H_32_	272.2493	2	273.2565	257.23, 161.13, 105.07	ND	ND	✓	ND	✓	ND	✓	✓

Note: RT, retention time; MW, molecular weight; MSI, Metabolomics Standards Initiative confidence level; CI, Ciamis accession; BOR, Bogor red accession; BOW, Bogor white accession; SI, Sukabumi accession; EtOAc, ethyl acetate extract; EtOH, ethanol extract; ✓, detected; ND, not detected.

**Table 3:** Predicted hub proteins identified from the protein–protein interaction network of the overlapping putative target genes.

No.	Proteins	PDB ID	Gene	Degree
1	Serine/threonine-protein kinase *AKT1*	6HHJ	*AKT1*	24
2	Hypoxia-inducible factor 1-alpha (HIF-1α)	3KCX	*HIF1A*	23
3	Cyclin D1 (*CCND1*)	2W96	*CCND1*	23
4	Epidermal growth factor receptor (*EGFR*)	8HV2	*EGFR*	22
5	B-cell lymphoma 2 protein (BCL-2)	8HTS	*BCL2*	20
6	Estrogen receptor alpha (ERα/*ESR1*)	9BU1	*ESR1*	19
7	Glyceraldehyde-3-phosphate dehydrogenase (*GAPDH*)	1U8F	*GAPDH*	18
8	Transcription factor AP-1 subunit Jun (c-Jun)	1JNM	*JUN*	16
9	Voltage-gated sodium channel NaV1.5 alpha subunit (*SCN5A*)	7DTC	*SCN5A*	15
10	Mechanistic target of rapamycin kinase (mTOR)	5H64	*MTOR*	14

**Table 4:** Physicochemical properties, drug-likeness screening, predicted ADMET profiles, and acute oral toxicity of the 15 terpenoid compounds and doxorubicin reference compound.

No.	Metabolites	MW (g/mol)	LogP	HBD	HBA	Lipinski	GI Absorption	BBB Permeability	CYP1A2 Inhibitor	CYP2C19 Inhibitor	CYP2C9 Inhibitor	CYP2D6 Inhibitor	CYP3A4 Inhibitor	Skin Permeability log Kp (cm/s)	Predicted LD50 (mg/kg)	Predicted Toxicity Class
1	α-Pinene-2-oxide	152.23	2.40	0	1	Yes	High	Yes	No	No	No	No	No	−5.72	5000	5
2	Thymol	150.22	2.80	1	1	Yes	High	Yes	Yes	No	No	No	No	−4.87	640	4
3	Pulegone	152.23	2.61	0	1	Yes	High	Yes	No	No	No	No	No	−5.04	470	4
4	*p*-Cymene	134.22	3.50	0	0	Yes	Low	Yes	No	No	No	Yes	No	−4.21	3	1
5	Norcamphor	110.15	1.47	0	1	Yes	High	Yes	No	No	No	No	No	−6.26	775	4
6	L-(−)-carvone	150.22	2.44	0	1	Yes	High	Yes	No	No	No	No	No	−5.29	1640	4
7	Kaur-16-ene	272.47	5.78	0	0	One violation: LogP > 5	Low	No	Yes	Yes	Yes	No	No	−3.06	5000	5
8	D-(+)-camphor	152.23	2.37	0	1	Yes	High	Yes	No	No	No	No	No	−5.67	775	4
9	Citral	152.23	2.71	0	1	Yes	High	Yes	No	No	No	No	No	−5.08	500	4
10	Carvone	150.22	2.44	0	1	Yes	High	Yes	No	No	No	No	No	−5.29	1640	4
11	(E,E)-α-farnesene	204.35	4.96	0	0	Yes	Low	No	Yes	No	Yes	No	No	−3.20	3650	5
12	(2E,6E)-farnesol	222.37	4.96	1	1	Yes	Low	No	Yes	No	Yes	No	No	−3.20	5000	5
13	1,8-Cineole	154.25	2.67	0	1	Yes	High	Yes	No	No	No	No	No	−5.30	2480	5
14	(+)-Alantolactone	232.32	3.19	0	2	Yes	High	Yes	No	Yes	Yes	No	No	−5.32	5000	5
15	(+)-Limonene	136.23	3.37	0	0	Yes	Low	Yes	No	No	Yes	No	No	−3.89	4400	5
16	Doxorubicin (reference compound)	543.52	0.52	6	12	No	Low	No	No	No	No	No	No	−8.71	205	3

Note: MW, molecular weight; LogP, octanol–water partition coefficient; HBD, hydrogen bond donor; HBA, hydrogen bond acceptor; ADMET, absorption, distribution, metabolism, excretion, and toxicity; GI absorption, predicted human intestinal absorption; BBB, blood–brain barrier; CYP, cytochrome P450; log Kp, predicted skin permeability coefficient; LD50, median lethal dose. Toxicity classes follow the Globally Harmonized System classification: Class 1, fatal if swallowed; Class 2, fatal if swallowed; Class 3, toxic if swallowed; Class 4, harmful if swallowed; Class 5, may be harmful if swallowed.

**Table 5:** Predicted binding energies (kcal mol^−1^) of the 15 terpenoid compounds and doxorubicin reference compound against ten selected protein targets.

No.	Metabolites	*GAPDH* (1U8F)	*ESR1* (9BU1)	*AKT1* (6HHJ)	*EGFR* (8HV2)	*SCN5A* (7DTC)	*BCL2* (8HTS)	*JUN* (1JNM)	*HIF1A* (3KCX)	*MTOR* (5H64)	*CCND1* (2W96)
1	α-Pinene-2-oxide	−5.8	−6.1	−5.5	−5.2	−6.3	−5.3	−3.3	−5.5	−6.6	−4.3
2	Thymol	−5.9	−6.0	−5.9	−5.1	−6.4	−5.8	−3.9	−6.0	−7.5	−5.0
3	Pulegone	−5.9	−6.2	−5.5	−5.1	−7.0	−6.2	−3.6	−6.4	−7.1	−5.1
4	*p*-Cymene	−5.4	−5.9	−5.2	−5.0	−6.8	−6.2	−3.6	−5.9	−7.8	−4.3
5	Norcamphor	−4.8	−5.0	−4.7	−4.3	−5.1	−4.8	−3.0	−4.8	−6.5	−4.5
6	L-(−)-carvone	−5.8	−6.0	−5.5	−5.3	−6.6	−5.9	−3.9	−6.0	−7.2	−4.9
7	Kaur-16-ene	−9.1	−9.8 *	−8.1	−7.8 *	−9.7 *	−7.1	−4.5	−8.2	−10.4	−6.6 *
8	D-(+)-camphor	−5.7	−5.8	−5.6	−5.3	−6.3	−5.9	−3.3	−5.5	−6.6	−4.5
9	Citral	−5.1	−5.3	−5.1	−4.9	−5.6	−5.5	−3.5	−5.7	−7.0	−4.6
10	Carvone	−5.8	−6.0	−5.5	−5.3	−6.6	−5.9	−3.9	−6.0	−7.2	−4.9
11	(E,E)-α-farnesene	−5.9	−6.6	−5.4	−5.5	−6.5	−6.7	−3.9	−6.6	−7.7	−5.3
12	(2E,6E)-farnesol	−6.1	−6.5	−5.9	−5.5	−7.0	−5.8	−3.8	−6.4	−7.2	−5.2
13	1,8-Cineole	−5.6	−5.2	−4.6	−5.2	−5.8	−5.6	−3.3	−5.2	−6.6	−4.4
14	(+)-Alantolactone	−8.7	−8.2	−7.6	−7.0	−8.8	−6.9	−4.5	−7.9	−9.2	−5.7
15	(+)-Limonene	−5.4	−5.7	−5.1	−4.9	−6.4	−5.7	−3.5	−5.7	−7.7	−4.6
16	Doxorubicin (reference compound)	−9.1	−6.7	−9.7	−7.3	−9.1	−7.4	−6.4	−8.5	−11.4	−6.1

Note: Binding energies are expressed in kcal mol^−1^. More negative docking scores indicate more favorable predicted ligand–protein interactions under the applied docking protocol. These values represent computational docking predictions and do not directly demonstrate pharmacological potency or target engagement. The asterisk (*) indicates terpenoid–protein pairs showing a more favorable predicted docking score than the doxorubicin reference compound under the same docking protocol.

## Data Availability

The original contributions presented in this study are included in the article/supplementary material. Further inquiries can be directed to the corresponding authors.

## Supplementary Materials

The following supporting information can be downloaded at: https://www.mdpi.com/article/10.3390/plants15162538/s1.
