# Supplementary material for: Secondary Metabolite Profiling, Antiproliferative Evaluation, and Terpenoid Prioritization of Indonesian Cardamom (Amomum compactum) Accessions
Source: Plants (Basel). 2026 Aug 21;15(16):2538. doi: 10.3390/plants15162538 (PMC13516252; doi:10.3390/plants15162538)
Supplement: Supplementary file 1 [file plants-15-02538-s001.zip › Table S1.pdf]

Table S1. Molecular docking interaction profiles of terpenoid compounds identified from four Indonesian cardamom accessions with ten breast cancer-related protein targets.

No.	GAPDH (1U8F)			
	Metabolites	Lowest Docking Score	Interaction	
			Hydrophobic	Hydrogen Bond
1	α-Pinene-2-oxide	-5.8	THR237(A), GLN204(B), THR237(C), ALA238(C), LEU203(B), GLN204(D), LEU203(D), ALA238(A), ASN239(C), ASN287(C)	ALA238(C)
2	Thymol	-5.9	ALA238(C), LEU203(D), ASN287(C), THR52(C), ASN239(C), THR237(C), PRO236(C), PRO236(D), LEU203(B), ALA238(A), THR237(A), GLN204(B)	GLN204(D)
3	Pulegone	-5.9	GLN204(B), LEU203(B), THR237(C), ASN239(C), SER284(C), ALA238(A), GLN204(D), LEU203(D), THR237(A)	ALA238(C)
4	p-cymene	-5.4	LEU203(D), GLN204(D), THR237(C), ALA238(C), GLN204(B), ASN287(A), ASN239(A), THR237(A), LEU203(B), ALA238(A)	-
5	Norcamphor	-4.8	ASN239(C), GLN204(B), GLN204(D), THR237(C), PRO236(C), LEU203(B), LEU203(D)	ALA238(C)
6	L-(-)-Carvone	-5.8	SER284(A), ASN239(A), THR237(A), GLN204(D), LEU203(D), LEU203(B), ALA238(C), THR237(C), GLN204(B), ASN287(A)	ALA238(A)
7	Kaur-16-ene	-9.1	ASN287(C), SER284(C), GLN204(D), ALA238(A), ASN239(A), VAL240(A), SER283(A), SER284(A), GLN204(B), SER51(C), THR52(C), ASN239(C), ALA238(C)	-
8	Isotretinoin	-7.6	HIS111(A), PRO36(A), ASN34(A), PRO82(A), ASN9(A), ASP35(A), THR99(A), SER98(A), GLY12(A), ARG13(A), GLY190(D), ALA183(A), PRO191(D), VAL101(A), ARG80(A), GLU79(A), PHE102(A), PHE37(A), ILE85(A)	-
9	D-(+)-Camphor	-5.7	THR52(C), ALA238(C), GLN204(B), GLN204(D), ASN239(C), SER51(A)	ASN287(C), SER284(C)
10	Citral	-5.1	THR237(A), THR273(C), LEU203(B), LEU203(D), ALA238 (C), GLN204(B), GLN204(D), ASN239(C), SER284(C)	ALA238(A), ASN239(A)

11	Carvone	-5.8	THR52(A), THR237(A), LEU203(B), LEU203(D), ALA238(A), ALA238(C), GLN204(B), GLN204(D), ASN287(A)	ALA238(A), ASN239(A)
12	(E, E)-alpha-Farnesene	-5.9	THR237(A), ASN239(A), SER284(C), ASN239(C), GLN204(B), GLN204(D), THR237(C), ALA238(A), ALA238(C), LEU203(B), LEU203(D)	-
13	(2E,6E)-Farnesol	-6.1	GLN204(B), THR237(A), ASN287(A), ALA238(C), GLN204(D), LEU203(B), THR237(C), LEU203(D), PRO236(B), PRO236(A)	ASN239(A)
14	1,8-Cineole	-5.6	THR25 (A), THR237(A), LEU203(B), LEU203(D), ALA238(A), GLN204(B), GLN204(D), ASN239(A)	ALA238 (A)
15	(+)-Alantolactone	-8.7	THR52(A), THR237(A), THR237(C), LEU203(B), LEU203(D), ALA238(A), ALA238(C), GLN204(B), GLN204(D), ASN287(A),	ALA238(A), ASN239(A)
16	(.+-.)-Limonene	-5.4	GLU79(A), ASN34(A), ASN9(A), GLY10(A), VAL101(A), THR99(A), PHR102(A), ARG80(A), PRO36(A), PHE37(A)	-
17	Doxorubicin	-9.1	SER98(A), ALA123(A), CYS152(A), ALE14(A), THR184(A), ASN316(A), GLU317(A), THR182(A), VAL188(D), ASP189(D), GLY190(D), ALA183(A), GLY12(A), PRO191(D), GLY190(D), ARG16(A), ILE38(A), ASP35(A), PHE37(A), ARG13(A)	ALA183(A), THR99(A), SER122(A)

No.	ESR1 (9BU1)			
	Metabolites	Lowest Docking Score	Interaction	
			Hydrophobic	Hydrogen Bond
1	α-Pinene-2-oxide	-6.1	ALA350(B), LEU349(B), LEU346(B), PHE404(B), LEU387(B), LEU384(B), LEU391(B), MET388(B), TRP383(B), LEU525(B), GLU353(B)	-
2	Thymol	-6.0	TRP393(B), ILE326(B), PHE445(B), ARG394(B), PRO324(B), LEU387(B), GLU323(B), LYS449(B), GLY390(B), ILE386(B), MET357(B), LEU327(B), PRO325(B)	GLU353(B)
3	Pulegone	-6.2	PRO324(B), TRP393(B), LEU387(B), MET357(B), LEU327(B), PRO325(B),	-

			ILE326(B), ARG394(B), GLU323(B), PHE445(B), LYS449(B), GLY390(B), ILE386(B), GLU353(B), HIS356(B)	
4	p-cymene	-5.9	PRO324(B), ARG394(B), PHE445(B), TRP393(B), ILE326(B), PRO325(B), HIS356(B), LEU387(B), MET357(B), ILE386(B), LYS449(B), GLY390(B)	-
5	Norcamphor	-5.0	LEU387(B), PRO324(B), MET357(B), PHE445(B), GLY390(B), ILE386(B), GLU353(B), ARG394(B)	LYS449(B)
6	L-(-)-Carvone	-6.0	PHE404(B), LEU525(B), LEU346(B), LEU391(B), MET388(B), LEU387(B), ALA350(B), TRP383(B), LEU384(B), GLU353(B), ARG394(B), THR347(B)	-
7	Kaur-16-ene	-9.8	LEU391(B), PHE404(B), LEU349(B), LEU387(B), ALA350(B), LEU346(B), LEU525(B), HIS(524(B), ILE424(B), GLU353(B), MET343(B), MET421(B), LEU384(B), MET388(B), LEU428(B), ARG394(B)	-
8	Isotretinoin	-7.6	ALA350(B), LEU346(B), LEU349(B), PHE404(B), LEU384(B), LEU391(B), MET388(B), LEU387(B), MET343(B), THR347(B), LEU525(B), GLU353(B), ARG394(B), LEU428(B), ASP(351(B)	-
9	D-(+)-Camphor	-5.8	LEU346(B), LEU387(B), LEU391(B), PHE404(B), MET388(B), ALA350(B), LEU525(B), LWU384(B), TRP383(B), GLU353(B)	-
10	Citral	-5.3	GLU323(B), GLY390(B), PHE445(B), LYS449(B), LEU387(B), LEU327(B), HIS356(B), PRO325(B), ARG394(B), ILE326(B), PRO324(B), GLU353(B), MET357(B), ILE386(B)	-
11	Carvone	-6.0	PHE404(B), LEU525(B), ALA350(B), LEU(346), LEU391(B), MET388(B), LEU387(B), TRP383(B), LEU384(B), GLU353(B), ARG394(B), THR347(B)	-
12	(E, E)-alpha-Farnesene	-6.6	LEU384(B), PHE404(B), LEU391(B), LEU387(B), LEU349(B), ALA350(B), LEU346(B), MET388(B), LEU428(B), GLU353(B), MET343(B), THR347(B), ARG394(B), LEU525(B), ILE424(B)	-
13	(2E,6E)-Farnesol	-6.5	MET421(B), PHE425(B), LEU428(B), LEU387(B), PHE404(B), LEU346(B), ALA350(B), MET388(B), LEU391(B),	LEU387(B)

			LEU525(B), ILE424(B), LEU402(B), ARG394(B), LEU384(B), MET343(B), GLU353(B)	
14	1,8-Cineole	-5.2	PHE404(B), LEU346(B), ALA350(B), LEU428(B), MET388(B), LEU(384), GLU353(B), LEU391(B), LEU349(B)	LEU387(B), ARG394(B)
15	(+)-Alantolactone	-8.2	ALA350(B), LEU349(B), LEU387(B), LEU391(B), LEU346(B), PHE404(B), LEU525(B), MET343(B)	THR347(B)
16	(.+-.)-Limonene	-5.7	LEU346(B), LEU525(B), ALA350(B), LEU387(B), LEU391(B), MET388(B), PHE 404(B), GLU353(B), ARG394(B), LEU384(B), THR347(B)	-
17	Doxorubicin	-6.7	MET388(B), LEU428(B), ILE424(B), LEU525(B), LEU346(B), ALA350(B), THR347(B), MET343(B), TRP383(B), LEU384(B), LEU387(B), LEU391(B), GLU353(B), PHE404(B), MET421(B), HIS524(B), PHE425(B)	THR347 (B)

No.	AKT (6HHJ)			
	Metabolites	Lowest Docking Score	Interaction	
			Hydrophobic	Hydrogen Bond
1	α-Pinene-2-oxide	-5.5	VAL270, ASP292(A), TYR272(A), VAL271(A), TRP80, THR81(A), GLN79(A), LYS179(A)	THR82(A)
2	Thymol	-5.9	LYS284(A), ASP283(A), GLU432(A), ALA230(A), TYR229(A), TYR175(A), ARG174(A), GLU228(A)	ASN231(A)
3	Pulegone	-5.5	TRP80(A), TYR272(A), ILE84(A), GLN79(A), LYS179(A), ASP292(A)	THR82(A)
4	p-cymene	-5.2	ILE84(A), ARG273(A), GLU17(A), ASN54(A), TYR326(A), GLN79(A), ARG86(A), GLU85(A), CYS296(A)	-
5	Norcamphor	-4.7	GLN79(A), LYS179(A), ASP292(A), TRP80(A), TYR272(A), VAL270(A)	THR81(A), THR82(A)
6	L-(-)-Carvone	-5.5	ARG174(A), TYR176(A), ALA230(A), LYS284(A), GLU228(A), TYR229(A), GLU432(A), ASN231(A), ASP283(A)	TYR175(A)
7	Kaur-16-ene	-8.1	TYR272(A), TRP80(A), VAL270(A), LEU264(A), ILE84(A), THR82(A), GLN79(A), TYR18(A), ARG273(A), ASP274(A), ASP292(A)	-

8	Isotretinoin	-8.1	ILE84(A), ARG86(A), ARG273(A), VAL270(A), TYR272(A), GLU17(A), TYR326(A), ASN54(A), ASP274(A), ASP292(A), TRP80(A), VAL271(A), GLN79(A), CYS296	GLU85(A)
9	D-(+)-Camphor	-5.6	VAL270(A), TYR272(A), VAL271(A), GLN79(A), THR81(A), TRP80(A), ASP292(A), LYS179(A)	THR82(A)
10	Citral	-5.1	VAL270(A), TYR272(A), ASN54(A), ARG273(A), GLN79(A), VAL271(A), LYS179(A), TRP80(A), ASP292(A)	THR80(A), THR82(A)
11	Carvone	-5.5	ARG174(A), TYR176(A), ALA230(A), LYS284(A), TYR229(A), GLU432(A), ASN231(A), ASP283(A), GLU228(A)	TYR175(A)
12	(E, E)-alpha-Farnesene	-5.4	TYR272(A), VAL270(A), TRP80(A), GLY294(A), ASP274(A), PHE293(A), LYS179(A), ILE84(A), ARG273(A), VAL271(A), ASN54(A), THR81(A), GLN79(A), ASP292(A), THR82(A)	-
13	(2E,6E)-Farnesol	-5.9	VAL270(A), TYR272(A), ARG273(A), TRP80(A), THR82(A), THR81(A), ILE84(A), VAL271(A), ASN54(A), ASP274(A), ASP292(A), LYS179(A)	GLN79(A)
14	1,8-Cineole	-4.6	ALA230(A), LYS284(A), GLU432(A), GLU228(A), ASN231(A), ASP283(A), LYS289(A), ARG174(A)	TYR229(A), TYR175(A)
15	(+)-Alantolactone	-7.6	TYR272(A), TRP80(A), VAL270(A), GLY294(A), ASP292(A), THR291(A), ASP274(A), GLN79(A), THR81(A)	LYS179(A), THR82(A)
16	(.+-.)-Limonene	-5.1	LYS284(A), TYR176(A), GLU432(A), TYR175(A), GLU228(A), TYR229(A), ALA230(A), ASN231(A), ASP283	-
17	Doxorubicin	-9.7	CYS296(A), ASP274(A), THR82(A), VAL270(A), LEU295(A), GLU85(A), ARG86(A), LYS20(A), GLU17(A), CYS310(A), LYS297(A), TYR18(A), GLY294(A), LYS179(A), ASP292(A), THR81(A), TRP80(A), GLN79(A), ASN54(A), VAL271(A), ARG273(A), ILE84(A)	ASP274(A), CYS296(A)

No.	EGFR (8HV2)			
	Metabolites	Lowest Docking Score	Interaction	
			Hydrophobic	Hydrogen Bond

1	α-Pinene-2-oxide	-5.2	LYS56(A), VAL37(A), LEU99(A), LEU155(A), ALA54(A), GLU73(A), MET77(A), THR101(A)	THR165(A)
2	Thymol	-5.1	PRO83(A), ARG87(A), ALA324(A), ASP325(A), ASP81(A), LEU89(A), CYS86	LYS163(A), VAL85(A), ASN82(A)
3	Pulegone	-5.1	ALA324(A), ARG87(A), LEU89(A), PRO83(A), LEU328(A), ASP81(A), ASN82(A), LYS163(A), ASP325(A), GLN102	ARG87(A)
4	p-cymene	-5.0	ALA324(A), ARG87(A), ASP325(A), LEU89(A), ASP81(A), PRO83(A), LYS163(A), ASN82(A), GLN102(A), TYR327(A), LEU328	-
5	Norcamphor	-4.3	ARG59(A), ALA66(A), SER63(A), LEU58(A), PHE34(A), ALA33	GLU60(A), ARG59
6	L-(-)-Carvone	-5.3	PRO83(A), LEU328(A), ARG87(A), LEU89(A), ALA324(A), ASP325(A), ASP81(A), ASN82(A), CYS8(A), VAL85(A)	ARG87(A), LYS163(A)
7	Kaur-16-ene	-7.8	LEU155(A), VAL37(A), LYS56(A), LEU99(A), ALA54(A), LEU29(A), GLY107(A), THR165(A), GLU73(A), MET77(A), THR101(A), LEU103(A), MET104(A)	-
8	Isotretinoin	-7.4	LEU29(A), VAL37(A), ALA54(A), LYS56(A), CYS86(A), LEU103(A), LEU155(A), GLY107(A), PHE106(A), PRO105(A), LEU312(A), THR165(A), THR101(A), MET77(A), GLU73(A), LEU99(A)	MET104
9	D-(+)-Camphor	-5.3	ALA54(A), LYS56(A), VAL37(A), LEU155(A), LEU99(A), GLU73(A), MET77(A), THR101(A), ASP166(A), CYS86(A)	THR165
10	Citral	-4.9	ALA54(A), LEU99(A), LYS56(A), VAL37(A), LEU155(A), ILE55(A), THR101(A), MET77(A), GLU73(A), LEU29(A), LEU103(A)	MET104(A)
11	Carvone	-5.3	PRO85(A), LEU328(A), ARG87(A), LEU89(A), ALA324(A), ASP325(A), ASP81(A), ASN83(A), CYS86(A), VAL85(A), GLN102(A)	ARG87(A), LYS163(A)
12	(E, E)-alpha-Farnesene	-5.5	LEU99A(A), LYS56(A), VAL37(A), LEU29(A), MET104(A), ALA54(A), LEU155(A), THR101(A), THR165(A), LEU103(A), GLY107(A)	-

13	(2E,6E)-Farnesol	-5.5	LYS56(A), VAL37(A), LEU155(A), LEU29(A), ALA54(A), MET104(A), THR165(A), LEU103(A), GLY107(A), CYS108(A), LEU99(A), ILE55(A), ILE100(A)	THR101(A), ALA54(A)
14	1,8-Cineole	-5.2	LEU99(A), LEU155(A), VAL37(A), LYS56(A), CYS86(A), ALA54(A), THR101(A), MET77(A), GLU73(A), ILE55(A), THR165(A)	-
15	(+)-Alantolactone	-7.0	VAL37(A), ALA54(A), LEU29(A), LEU155(A), LYS56(A), MET77(A), LEU99(A), GLU73(A), THR101(A), MET104(A)	ASP166(A)
16	(.+-.)-Limonene	-4.9	PRO83(A), ARG87(A), LEU89(A), ALA324(A), LEU328(A), ASP81(A), LYS163(A), ASN82(A), TYR327(A), GLN102(A), ASP325(A)	-
17	Doxorubicin	-7.3	LEU844(A), ALA743(A), CYS775(A), MET766(A), VAL726(A), ASP855(A), LYS745(A), GLU758(A), SER720(A), GLY724(A), ILE759(A), GLY857(A), THR790(A)	GLU762(A), LYS745(A), GLU758(A)

No.	SCN5A (7DTC)			
	Metabolites	Lowest Docking Score	Interaction	
			Hydrophobic	Hydrogen Bond
1	α-Pinene-2-oxide	-6.3	PHE1418(A), LEU895(A), ILE1455(A), PHE1459(A), ILE1454(A), LEU846(A), CYS8896(A), VAL930(A), SER1458(A), PHE892(A)	-
2	Thymol	-6.4	ILE1756(A), LEU1462(A), PHE1465(A), PHE1760(A), THR1417(A), SER1710(A), LEU1413(A), ILE1757(A), TRP1345(A), THR1461(A)	-
3	Pulegone	-7.0	LEU1462(A), PHE1465(A), PHE1760(A), ILE1756(A), THR1417(A), SER1710(A), ILE1757(A), LEU1413(A), THR1461(A)	-
4	p-cymene	-6.8	PHE1465(A), PHE1760(A), ILE1756(A), ILE1757(A), SER1710(A), THR1417(A), LEU1462(A), THR1461(A)	-
5	Norcamphor	-5.1	VAL930(A), PHE1459(A), PHE1418(A), ILE1455(A), ILE1454(A), CYS896(A), PHE892(A), LEU895(A)	SER1458(A)

6	L-(-)-Carvone	-6.6	LEU1462(A), PHE1465(A), LEU1338(A), ILE1757(A), PHE1760(A), THR1417(A), ILE1756(A), LEU1413(A)	-
7	Kaur-16-ene	-9.7	LEU1338(A), LEU1342(A), TRP1345(A), PHE1465(A), LEU1462(A), PHE1760(A), LEU1413(A), ILE1757(A), CYS1341(A), THR1461(A), THR1417(A), TYH1409(A)	-
8	Isotretinoin	-9.6	PHE1760(A), ILE1757(A), PHE1465(A), LEU1338(A), LEU1462(A), TRP1345(A), SER1710(A), ILE1756(A), CYS1341(A), THR1461(A), THR1417(A), THR1709(A), PHE402(A), VAL1763(A)	THR1708(A), SER1759(A), PHE1760(A)
9	D-(+)-Camphor	-6.3	PHE1760(A), LEU1462(A), ILE1757(A), LEU1413(A), PHE1465(A), THR1417(A), ILE1756(A), TRP1345(A), THR1461(A)	-
10	Citral	-5.6	LEU1462(A), ILE1757(A), PHE1760(A), PHE1465(A), THR1461(A), CYS1341(A), LEU138(A), LEU1413(A), ILE1756(A), THR1417(A)	-
11	Carvone	-6.6	LEU1462(A), PHE1465(A), LEU1338(A), ILE1757(A), PHE1760, ILE1756(A), THR1417(A)	-
12	(E, E)-alpha-Farnesene	-6.5	PHE1760(A), ILE1757(A), LEU1462(A), LEU1338(A), ILE1756(A), PHE1465(A), TYR1409(A), LEU1342(A), CYS1341(A), TRP1345(A), LEU1413(A), THR1461(A)	
13	(2E,6E)-Farnesol	-7.0	LEU1342(A), TYR1409(A), TRP1345(A), LEU1338(A), PHE1465(A), LEU1462(A), PHE1760(A),THR1417(A), ILE1756(A), ILE1757(A), LEU1413(A), THR1561(A)	-
14	1,8-Cineole	-5.8	PHE1465(A), LEU1462(A),PHE1760(A), THR1417(A), ILE1756(A), SER1710(A), THR1708(A), THR1709(A), VAL1763(A), ILE1757(A)	SER1759(V)
15	(+)-Alantolactone	-8.8	LEU1338(A), ILE1757(A), PHE1760(A), LEU1413(A), ILE1756(A), PHE1465(A), TRP1345(A), LEU1462(A), THR1461(A), CYS1341(A), THR1417(A)	-
16	(.+-.)-Limonene	-6.4	ILE1756(A), PHE1760(A), LEU1462(A), TRP1345(A), PHE1465(A), THR1417(A), THR1461(A), ILE1757(A), SER1710(A)	-
17	Doxorubicin	-9.1	ILE1757(A), PHE1760(A), THR1417(A), LEU1462(A),TRP1345(A), THR1461(A), LEU1413(A), ILE1756(A), SER1710(A), TYR1767(A), PHE402(A), GLN371(A),	LYS1419(A), SER1458(A)

			PHE1459(A), PHE934(A), VAL930(A), PHE1418(A), ALA1416(A)	

No.	CCND1			
	Metabolites	Lowest Docking Score	Interaction	
			Hydrophobic	Hydrogen Bond
1	α-Pinene-2-oxide	-4.3	VAL60(A), ILE59(A), MET56(A), LYS96(A), LEU99(A), GLN100(A)	-
2	Thymol	-5.0	LEU142(A), TRP150(A), VAL145(A), MET113(A), LYS112(A), GLU141(A), VAL109(A), LU152(A)	GLU141(A)
3	Pulegone	-5.1	LEU142(A), VAL145(A), TRP150(A), VAL109(A), MWT113(A), LYS112(A), LEU152(A), GLU141(A)	-
4	p-cymene	-4.3	TRP150(A), LEU142(A), GLU141(A), VAL145(A), LYS112(A), VAL109(A), LEU152(A)	-
5	Norcamphor	-4.5	ALA187(A), CYS73(A), VAL77(A), GLU74(A), CYS68(A), LEU65(A), PRO79(A), GLU69(A), GLU75(A)	PHE78(A)
6	L-(-)-Carvone	-4.9	LEU142(A), TRP150(A), LYS11, GLU141(A), LEU152(A), VAL109(A)	-
7	Kaur-16-ene	-6.6	LYS72(A), ILE117(A), LEU119(A), LYS123(A), THR120(A), PRO118(A), GLN71(A), CYS73(A), GLU115(A), GLU74(A)	-
8	Isotretinoin	-5.8	HIS158(A), HIS163(A), MET31(A), VAL27(A), LEU6(A), GLU162(A), GLU74(A), GLU75(A), GLN 183(A), MET155(A), ASP159(A)	-
9	D-(+)-Camphor	-4.5	ILE59(A), VAL60(A), MET56(A), LEU99(A), LYS96(A), GLN100(A)	-
10	Citral	-4.6	LEU142(A), VAL145(A), TRP150(A), VAL109(A), LEU152(A), MET113(A), LYS112(A), GLU141(A)	-
11	Carvone	-4.9	VAL145(A), TRP150(A), LEU142(A), LYS112(A), VAL109(A), LEU152(A), GLU141(A)	LYS112(A)
12	(E, E)-alpha-Farnesene	-5.3	LYS112(A), VAL109(A), LEU152(A), TRP150(A), LEU142(A), LEU138(A), VAL145(A), GLU141(A), MET113(A)	-
13	(2E,6E)-Farnesol	-5.2	MET113(A), LEU152(A), LYS112(A), VAL109(A), VAL145(A), LEU142(A), TRP150(A), ASN151(A), GLU141(A)	ALA153(A), TRP159(A)

14	1,8-Cineole	-4.4	LYS96(A), ILE59(A), MET56(A), LEU99(A), VAL60(A), GLN100(A)	-
15	(+)-Alantolactone	-5.7	LEU6(A), VAL27(A), MET31(A), HIS163(A), ASP159(A), GLU162(A), CYS7(A), LEU23(A), ASN24(A)	LEU6(A)
16	(.+-.)-Limonene	-4.6	LYS112(A), LEU142(A), VAL145(A), VAL109(A), LEU152(A), GLU141(A)	-
17	Doxorubicin	-6.1	LYS114(A), ASP159(A), MET113(A), THR156(A), ALA153(A), MET155(A), LEY6(A), MET31(A), VAL27(A), HIS163(A), SER166(A), GLU162(A)	GLU76(A), ALA154(A)

No.	BCL2 (8HTS)			
	Metabolites	Lowest Docking Score	Interaction	
			Hydrophobic	Hydrogen Bond
1	α-Pinene-2-oxide	-5.3	TYR161(A), VAL107(A), ALA59(A), PHE157(A), ASP62(A), PHE63(A), GLY104(A)	-
2	Thymol	-5.8	PHE71(A), MET74(A), ALA108(A), PHE112(A), LEU96(A), VAL92(A), ASP70(A), TYR67(A), PHE63(A), PHE109(A)	-
3	Pulegone	-6.2	PHE71(A), TYR67(A), MET74(A), LEU96(A), ALA108(A), PHE112(A), VAL92(A), ASP70(A), PHE63(A), PHE109(A)	-
4	p-cymene	-6.2	PHE63(A), PHE71(A), TYR67(A), ALA108(A), VAL92(A), PHE112(A), LEU96(A), PHE109(A), MET74(A), ASP70(A)	-
5	Norcamphor	-4.8	TYR161(A), ALA59(A), VAL107(A), PHE63(A), GLY104(A), PHE157(A), TRP103(A)	-
6	L-(-)-Carvone	-5.9	ALA108(A), MET74(A), PHE112(A), LEU96(A), VAL92(A), PHE63(A), PHE71(A), TYR67(A), ASP70(A), PHE109(A)	-
7	Kaur-16-ene	-7.1	ALA59(A), ASP62(A), TYR161(A), VAL107(A), PHE63(A), GLY104(A), ARG66(A), TYR67(A), ASN102(A)	-
8	Isotretinoin	-6.8	MET74(A), PHE71(A), PHE63(A), TYR67(A), LEU96(A), ALA108(A), ARG105(A), GLU111(A), PHE112(A), ASP70(A)	-

9	D-(+)-Camphor	-5.9	VAL107(A), ALA59(A), TYR161(A), PHE63(A), ASP62(A), PHE157(A), TRP103(A), GLY104(A)	-
10	Citral	-5.5	LEU96(A), PHE71(A), MET74(A), TYR67(A), PHE63(A), ALA108(A), ASP70(A), PHE112(A), VAL92(A), GLU96(A)	-
11	Carvone	-5.9	PHE71(A), ALA108(A), LEU96(A), PHE112(A), MET74(A), VAL92(A), PHE63(A), TYR67(A), ASP70(A), PHE109(A)	-
12	(E, E)-alpha-Farnesene	-6.7	PHE112(A), VAL92(A), LEU96(A), ALA108(A), MET74(A), PHE71(A), TYR67(A), PHE63(A), ASP70(A), GLU96(A), THR91(A)	-
13	(2E,6E)-Farnesol	-5.8	PHE63(A), MET74(A), VAL92(A), PHE112(A), LEU96(A), PHE71(A), ALA108(A), GLN77(A), GLU73(A), ASP70(A), TYR67(A), GLI95(A)	-
14	1,8-Cineole	-5.6	TYR67(A), LEU96(A), PHE63(A), MET74(A), ALA108(A), PHE71(A), ASP70(A), PHE112(A)	
15	(+)-Alantolactone	-6.9	PHE63(A), PHE71(A), TYR67(A), ALA108(A), LEU96(A), MET74(A), ASP70(A)	-
16	(.+-.)-Limonene	-5.7	LEU96(A), PHE112(A), VAL92(A), ALA108(A), PHE63(A), MET74(A), TYR67(A), PHE71(A), ASP70(A), PHE109(A)	-
17	Doxorubicin	-7.4	GLU13(A), VAL36(A), PRO44(A), TYR9(A), ARG12(A), SER49(A), ARG40(A), LYS17(A), GLU37(A), ASN39(A), ALA43(A), GLU48(A)	ASP10(A), VAL36(A), THR47(A)

No.	JUN (1JNM)			
	Metabolites	Lowest Docking Score	Interaction	
			Hydrophobic	Hydrogen Bond
1	α-Pinene-2-oxide	-3.3	LYS273(A), ARG276(A), ARG272(A),SER269(A)	ARG272(A)
2	Thymol	-3.9	LYS273(A), ARG272(A), ARG276(A), SER269(A), ILE277(A)	-
3	Pulegone	-3.6	ARG272(A), ARG276(A), LYS273(A), SER269(A)	LYS273(A)
4	p-cymene	-3.6	LYS273(A), ARG272(A), ARG276(A), SER269(A), ARG270(A)	-
5	Norcamphor	-3.0	LYS258(A), ARG259(A), ALA255(A), ASN262(A)	ARG259(A)
6	L-(-)-Carvone	-3.9	ARG279(A), ARG272(A), LYS273(A), SER269(A)	ARG272(A)
7	Kaur-16-ene	-4.5	ALA255(A), LYS258(A), ARG259(A), ASN262(A)	-
8	Isotretinoin	-5.0	LYS273(A), ARG270(A), ALA266(A), ASN262(A), ARG276(A)	SER269(A), ARG272(A)
9	D-(+)-Camphor	-3.3	LYS273(A), ARG276(A), SER269(A)	ARG272(A)
10	Citral	-3.5	ARG270(A), LYS273(A), SER269(A), ARG276(A), ARG279(A)	ARG272(A)
11	Carvone	-3.9	ARG276(A), ARG272(A), LYS273(A), SER269(A)	ARG272(A)
12	(E, E)-alpha-Farnesene	-3.9	LYS258(A), ARG259(A), ARG263(A), ALA266(A), ARG270(A), ASN262(A), ALA255(A)	-
13	(2E,6E)-Farnesol	-3.8	ARG276(A), LYS273(A), SER269(A), ARG272(A), ARG270(A), ARG270(A)	LYS273(A)
14	1,8-Cineole	-3.3	LYS258(A), ARG259(A), ASN262(A), ALA255(A)	-
15	(+)-Alantolactone	-4.5	ARG270(A), LYS273(A), SER269(A)	ARG276(A), LYS273(A)
16	(.+-.)-Limonene	-3.5	ARG276(A), LYS273(A), SER269(A)	-

17	Doxorubicin	-6.4	LYS288(B), VAL284(A), LYS285(A), GLU281(A), LEU280(A), ARG276(A), LYS283(A), GLU282(A)	ARG279(A)

No.	HIF1A (3KCX)			
	Metabolites	Lowest Docking Score	Interaction	
			Hydrophobic	Hydrogen Bond
1	α-Pinene-2-oxide	-5.5	TRP296(A), LEU188(A), HIS199(A), TYR145(A), THR196(A), GLN147(A), ASN294(A), PHE207(A), LEU186(A), ILE281(A)	-
2	Thymol	-6.0	TYR102(A), ARG238(A), HIS199(A), ASP237(A), GLN147(A), THR196(A), PHE100(A), PRO197(A)	GLN239(A)
3	Pulegone	-6.4	TYR145(A), LEU188(A), PHE207(A), ILE281(A), HIS199(A), HIS279(A), ILE273(A), GLN147(A), LYS214(A), ASN294(A), ASN205(A), TRP296(A)	THR196(A)
4	p-cymene	-5.9	HIS199(A), ARG238(A), TYR102(A), GLN239(A), ASP237(A), THR196(A), GLN147(A)	-
5	Norcamphor	-4.8	LEU188(A), HIS199(A), ILE281(A), ASP201(A), HIS279(A), THR196(A), PHE207(A), ASN294(A), ILE273(A), TRP296(A)	ASN205(A)
6	L-(-)-Carvone	-6.0	HIS199(A), TYR102(A), ARG238(A), ASP201(A), THR196(A), GLN147(A), GLN239(A), ASP237(A)	ARG238(A)
7	Kaur-16-ene	-8.2	TRP296(A), LEU186(A), HIS199(A), LEU188(A), ASP201(A), ARG238(A), GLN147(A), THR196(A), TYR145(A)	-
8	Isotretinoin	-7.8	TYR145(A), ILE281(A), HIS279(A), LEU188(A), HIS199(A), LEU186(A), ASN294(A), TRP296(A), GLN203(A), GLN147(A), THR196(A), LYS214(A), PHE207(A)	THR183(A), SER184(A)
9	D-(+)-Camphor	-5.5	LEU188(A), HIS199(A), TRP296(A), LEU186(A), ILE281(A), HIS279(A), ASN294(A), GLN147(A), TYR145(A)	THR196(A)
10	Citral	-5.7	TYR145(A), LEU188(A), HIS199(A), TYR102(A), ASP201(A), GLN147(A), THR196(A), PHE100(A), GLN239(A)	ARG238(A)

11	Carvone	-6.0	HIS199(A), TYR102(A), ASP201(A), GLN147(A), THR196(A), GLN239(A), ASP237(A)	ARG238(A)
12	(E, E)-alpha-Farnesene	-6.6	TYR93(A), TYR102(A), TYR145(A), PHE100(A), GLN147(A), THR196(A), LEU188(A), ILE281(A), PHE207(A), LEU186(A), ASN294(A), HIS199(A), TRP296(A), HIS279(A), ASN205(A)	-
13	(2E,6E)-Farnesol	-6.4	TYR102(A), HIS199(A), LEU188(A), LEU186(A), HIS279(A), TRP296(A), ILE281(A), ASP201(A), THR196(A), GLN147(A), ASN294(A), PHE207(A)	-
14	1,8-Cineole	-5.2	ILE281(A), TRP296(A), LEU188(A), PHE207(A), LYS214(A), HIS199(A), ASN294(A), HIS279(A), LEU186(A), TYR145(A), TYR102(A), GLN147(A), PHE100(A)	THR196(A)
15	(+)-Alantolactone	-7.9	ILE281(A), HIS199(A), TYR102(A), ARG238(A), ASP201(A), LEU186(A), TRP296(A), GLN147(A), LEU188(A), TYRR145(A)	THR196(A)
16	(.+-.)-Limonene	-5.7	TRP296(A), PHE207(A), LEU188(A), TYR145(A), TYR102(A), GLN147(A), THR196(A), HIS279(A), HIS199(A), ILE281(A), ASN205(A), ILE273(A), ASN294(A)	-
17	Doxorubicin	-8.5	TYR102(A), TRP296(A), LEU186(A), THR149(A), LEU188(A), GLN203(A), HIS199(A), ASP201(A), LYS214(A), HIS279(A), PHE207(A), TYR145(A), ILE281(A), ILE273(A), ASN294(A), ASN205(A), PRO197(A), PHE100(A), GLN147(A), TYR93(A)	SER184(A), THR196(A)

No.	MTOR (5H64)			
	Metabolites	Lowest Docking Score	Interaction	
			Hydrophobic	Hydrogen Bond
1	α-Pinene-2-oxide	-6.6	LEU351(B), LEU147(B), PHE192(B), GLN109(B), TYR110(B), LEU84(B), CYS86(B), HIS149(B), TYR194(B), LEU106(B)	
2	Thymol	-7.5	ARG2317(A), ARG2316(A), TRP2313(A), LEU2303(A), TYR2320(A), LEU2346(A),	ASP2347(A)

			SER2307(A), GLY2351(A), ARG2348(A), LEU2299(A), ILE2353(A), LYS2352(A)	
3	Pulegone	-7.1	ALA210(B), PRO259(B), LYS169(B), LEU262(B), PHE206(B), ILE191(B), PHE263(B), VAL203(B), LYS207(B), ASN168(B), THR264(B)	-
4	p-cymene	-7.8	PHE1022(A), PHE979(A), VAL993(A), LEU1019(A), VAL1000(A), PHE997(A), HIS1026(A), GLY983(A), LEU982(A), ARG957(A), ARG960(A)	-
5	Norcamphor	-6.5	LEU1180(A), ILE1156(A), LEU1177(A), LYS1187(A), VAL1181(A), TYR1188(A), PHE1148(A), PHE1191(A), VAL1195(A), TYR1209(A), ILE1159(A), ILE1155(A)	-
6	L-(-)-Carvone	-7.2	-	-
7	Kaur-16-ene	-10.4	LEU106(B), ARG348(B), TYR194(B), LEU351(B), LEU84(B), PHE192(B), LEU147(B), PRO115(B), CYS86(B), LEU352(B), GLN109(B), TRP113(B), ALA415(B), GLN114(B), GLU411(B), HIS149(B)	-
8	Isotretinoin	-10.1	LYS132(B), PHE148(B)	ASN168(B)
9	D-(+)-Camphor	-6.6	-	GLN694(A)
10	Citral	-7.0	VAL993(A), PHE979(A), PHE997(A), VAL1000(A), PHE1022(A), LEU1019(A), MET1018(A), THR996(A), VAL1003(A), PHE1011(A), LEU964(A), THR976(A)	GLN1015(A)
11	Carvone	-7.2	PHE997(A), VAL1000(A), LEU1019(A), PHE1022(A), PHE979(A), LEU982(A), ARG957(A), VAL993(A), GLY983(A), ARG960(A), THR996(A)	HIS1026(A)
12	(E, E)-alpha-Farnesene	-7.7	ARG145(B), ALA82(B), LEU351(B), PRO115(B), PHE192(B), LEU106(B), CYS86(B), GLN109(B), TYR110(B), LEU84(B), GLN114(B), SER190(B), VAL146(B), ARG83(B), HIS149(B)	-
13	(2E,6E)-Farnesol	-7.2	LEU106(B), CYS86(B), PHE192(B), LEU351(B), PRO115(B), LEU147(B), LEU84(B), GLN109(B), TYR194(B), HIS149(B), GLU85(B), GLN114(B), GLU411(B), SER190(B), TYR110(B)	ARG348(B)
14	1,8-Cineole	-6.6	LEU84(B), LEU147(B), PHE192(B), LEU106(B), LEU351(B), HIS149(B), TYR194(B), TYR110(B), GLN109(B)	-
15	(+)-Alantolactone	-9.2	LEU84(B), PRO115(B), LEU147(B), PHE192(B), LWU351(B), GLU411(B),	-

			SER190(B), ARG83(B), GLN114(B), HIS149(B), LEU106(B), CYS86(B), GLN109(B), TYR110(B), TRP113(B)	
16	(.+-.)-Limonene	-7.7	PHE997(A), VAL993(A), VAL1000(A), LEU1019(A), PHE1022(A), LEU982(A), ARG957(A), PHE979(A), HIS1026(A), GLY983(A), ARG960(A), THR996(A)	-
17	Doxorubicin	-11.4	PHE148(B), VAL164(B), TYR150(B), PRO158(B), ASN168(B), LYS169(B), LEU262(B), ILE191(B), PHE206(B), ILE202(B), TRP87(B), ASP128(B), GLU85(B), VAL146(B), THR135(B), SER136(B), PRO189(B)	PHE167(B), LEU147(B), LYS132(B)
