# Supplementary material for: Secondary Metabolite Profiling, Antiproliferative Evaluation, and Terpenoid Prioritization of Indonesian Cardamom (Amomum compactum) Accessions
Source: Plants (Basel). 2026 Aug 21;15(16):2538. doi: 10.3390/plants15162538 (PMC13516252; doi:10.3390/plants15162538)
Supplement: Supplementary file 1 [file plants-15-02538-s001.zip › Table S2.pdf]

Table S2. Grid box coordinates and dimensions of binding sites in ten breast cancer-related protein targets used for molecular docking analysis

No.	Proteins	Center_x	Center_y	Center_z	Size_x	Size_y	Size_z
1	GAPDH (1U8F)	41.4809249111	42.3232851889	47.5647289911	43.5634498221	37.7523703778	36.8950645225
2	ESR1 (9BU1)	24.069745838	-43.7721976052	17.1167540414	26.7537490461	72.3396781101	16.273886631
3	AKT1 (6HHJ)	9.7149706437	-11.7717442257	-12.886318255	16.3971412874	21.5494884515	23.4956365101
4	EGFR (8HV2)	-2.52915314705	-26.8058782219	-50.6323017158	34.9764937059	20.9172435563	9.03420343156
5	SCN5A (7DTC)	134.296573382	128.497666037	127.161813908	65.0323957021	60.0546257459	21.7577473204
6	CCND1 (2W96)	17.1682397181	-4.64706171064	58.2331832463	57.1157736043	26.5228555258	28.0661540029
7	BCL2 (8HTS)	6.19261164442	12.2521605204	37.6495384856	18.0258232888	21.2160789592	26.1695071804
8	JUN (1JNM)	11.6528221729	11.5689583646	6.63873915086	54.420978947	54.420978947	56.8061204954
9	HIF1A (3KCX)	-23.2618180759	25.7963161447	5.59066995006	53.9099374524	29.9168532069	34.1790141028
10	MTOR (5H64)	145.058774833	154.66722109	193.888746416	74.9677424663	57.6033578199	17.1684928313
